# Supplementary material for: Cellular remodeling and JAK inhibition promote zygotic gene expression in the Ciona germline
Source: EMBO Rep. 2024 Apr 22;25(5):2188–201. doi: 10.1038/s44319-024-00139-0 (PMC11094015; doi:10.1038/s44319-024-00139-0)
Supplement: Supplementary file 1 — Appendix [file 44319_2024_139_MOESM1_ESM.pdf]

Table of contents

Appendix Figure S1	Page 2-3
Appendix Figure S2	Page 4
Appendix Figure S3	Page 5
Appendix Figure S4	Page 6
Appendix Figure S5	Page 7
Appendix Figure S6	Page 8
Appendix Figure S7	Page 9
Appendix Figure S8	Page 10
Appendix Table S1	Page 11
Appendix Table S2	Page 12

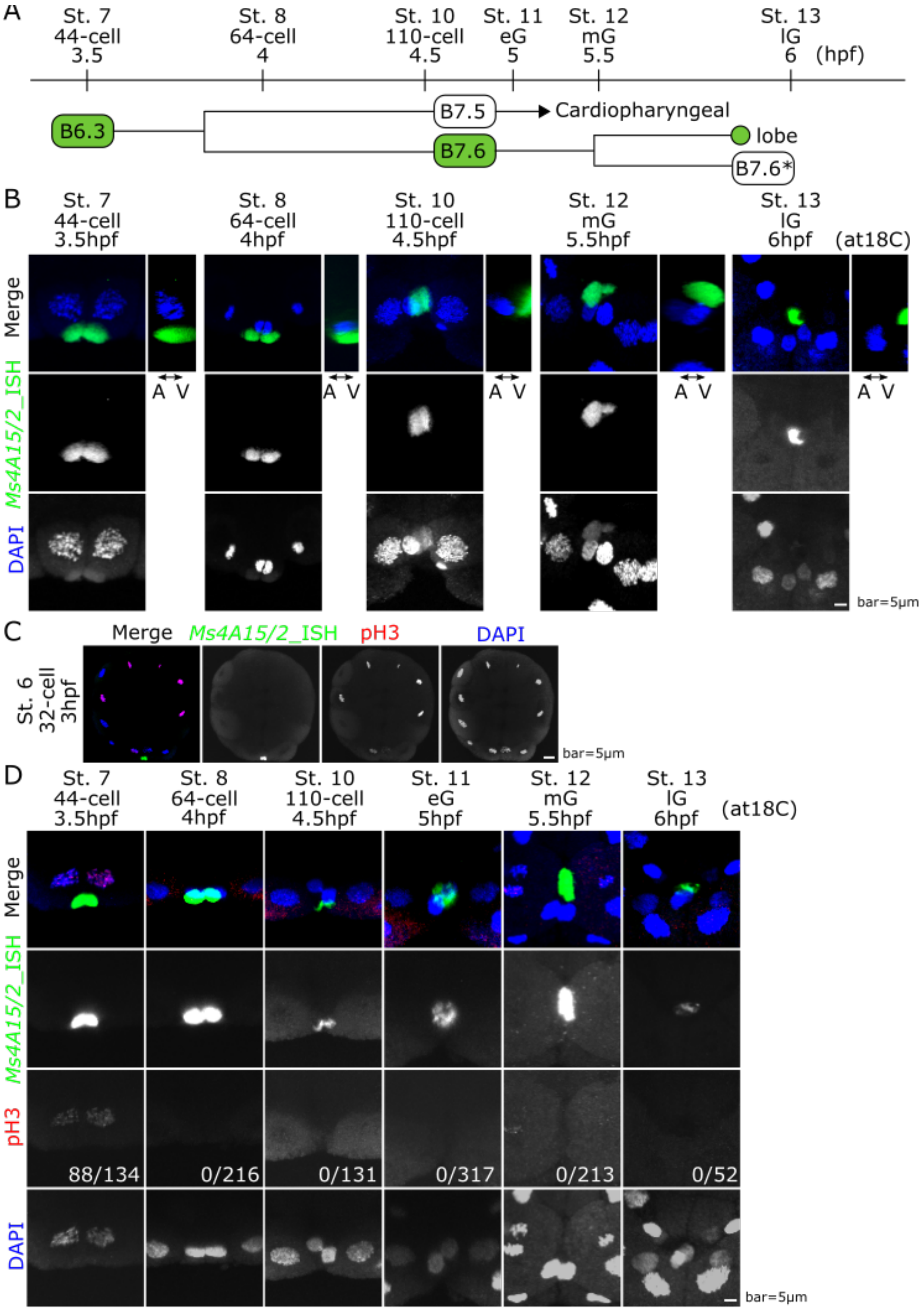

Appendix Figure S1. (A) Time scale of cell division from 32-cell stage to late gastrula stage in *Ciona* embryo. (B) Time series embryos from 44-cell stage to late gastrulation (lG) stage were stained by PGC marker *Ms4A15/2* by FISH. These pictures are vegetal view. The pictures on the side are lateral view. (C) The embryos at 32-cell stage were stained by both PGC marker *Ms4A15/2* by FISH and anti-pH3 antibody. (D) Time series embryos from 44-cell stage to late gastrulation (lG) stage were stained by both PGC marker *Ms4A15/2* by FISH and anti-pH3 antibody. Numbers shows the pH3 signal positive in B6.3 or B7.6 cells

out of total embryos. These pictures are vegetal view. Nuclei were stained with DAPI showed in blue. Scale bars are 5 µm on B-D.

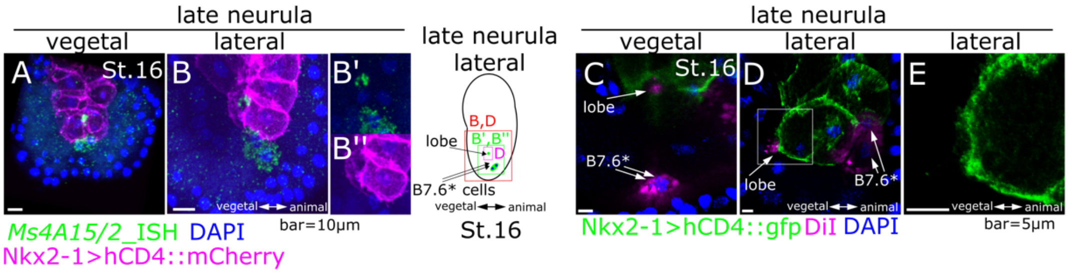

Appendix Figure S2. (A-B) Both lobe and B7.6* cells were stained by ISH for *MS4a15/2* gene, and cell membranes of endodermal cells were stained by Nkx2-1>hCD4::mCherry at the late neurula stage, St. 16; vegetal (A) and lateral (B) views. (B') Green and blue channels of B. (B'') Magenta and blue channels of B. Scale bars are 10 μm on A-B. (C-D) Endodermal cell membrane was stained by Nkx2-1>hCD4::gfp in addition to DiI labeling for B7.6 cells. Scale bars are 5 μm on A-B.

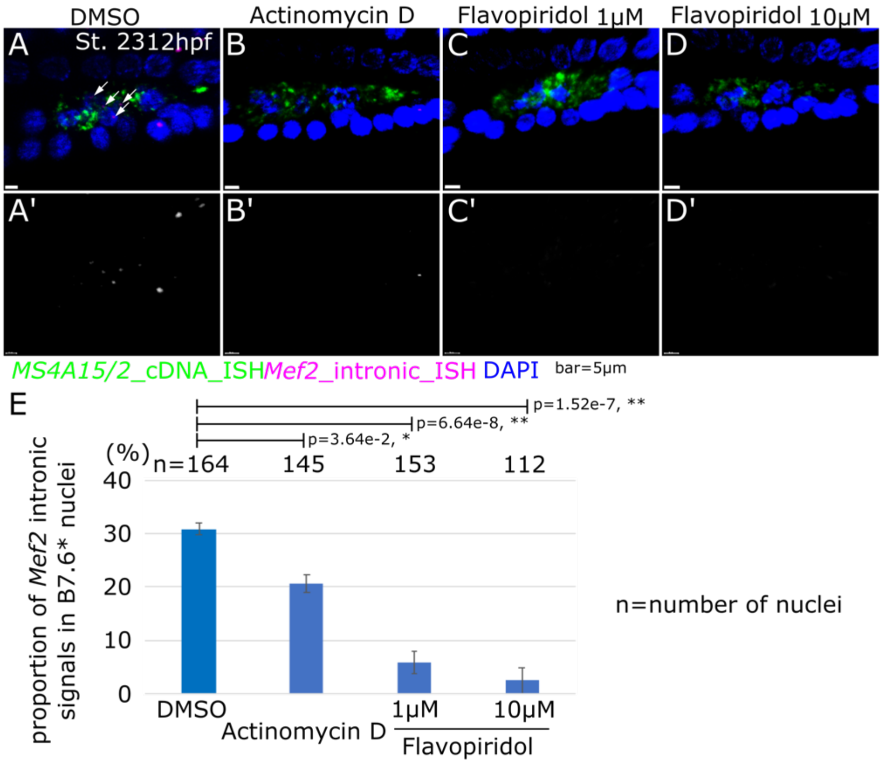

Appendix Figure S3. (A-D') Nascent expression of *Mef2* in embryos treated by: DMSO (A), Actinomycin D (B), 1μM (C) and 10μM (D) Flavopiridol. A'-D' shows black and white image of the magenta channel. White arrows indicate the dotted signals of nascent *Mef2* expression in nuclei of B7.6* cells. Scale bars are 5 μm on A-D. (E) Proportion of *Mef2* signals in nuclei in B7.6* cells. Error bars indicate standard error on E. p-value was calculated by z-test on E. p>0.05; N.S, 0.05>p>0.01; *, 0.01>p; **.

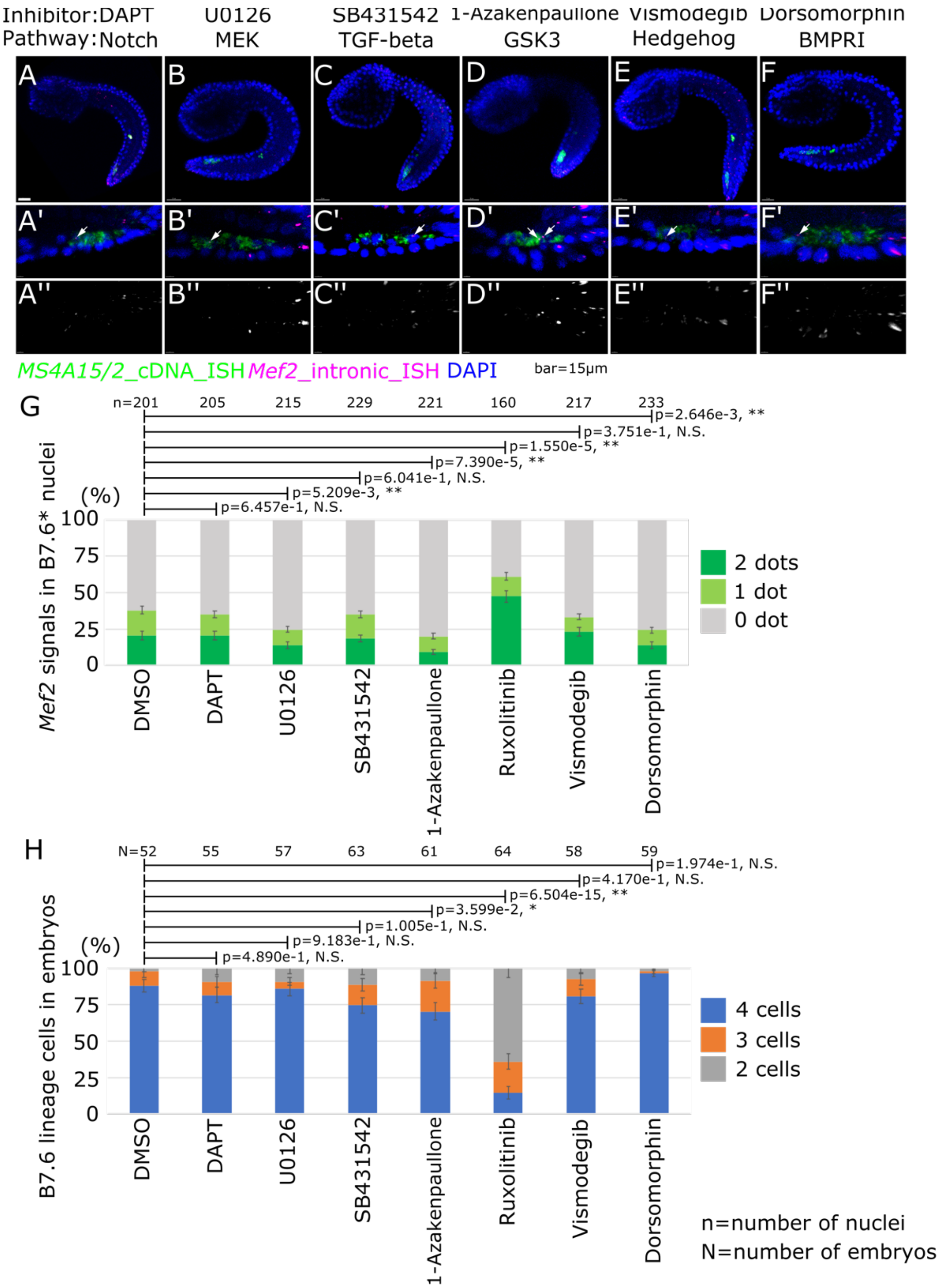

Appendix Figure S4. (A-F) ISH was done with *Mef2* intronic probes under each pharmacological inhibitor treatment. White arrows indicate the dotted signals of nascent *Mef2* expression in nuclei of B7.6* cells. Scale bar=15μm. (A'-F') Magnification images around B7.6* cells. (A''-F'') Black and white image of the magenta channel of A'-F', respectively. (G) Proportion of *Mef2* signals in nuclei of B7.6* cells under each pharmacological inhibitor treatment. The proportion of signal positive nuclei was shown in y-axis. (H) Proportion of cell numbers of B7.6* cells in embryos under each pharmacological inhibitor treatment. The proportion of the number of cells in the embryos was shown in y-axis. Error bars indicate standard error on G and H. p-value was calculated by z-test on G and H. p>0.05; N.S, 0.05>p>0.01; *, 0.01>p; **.

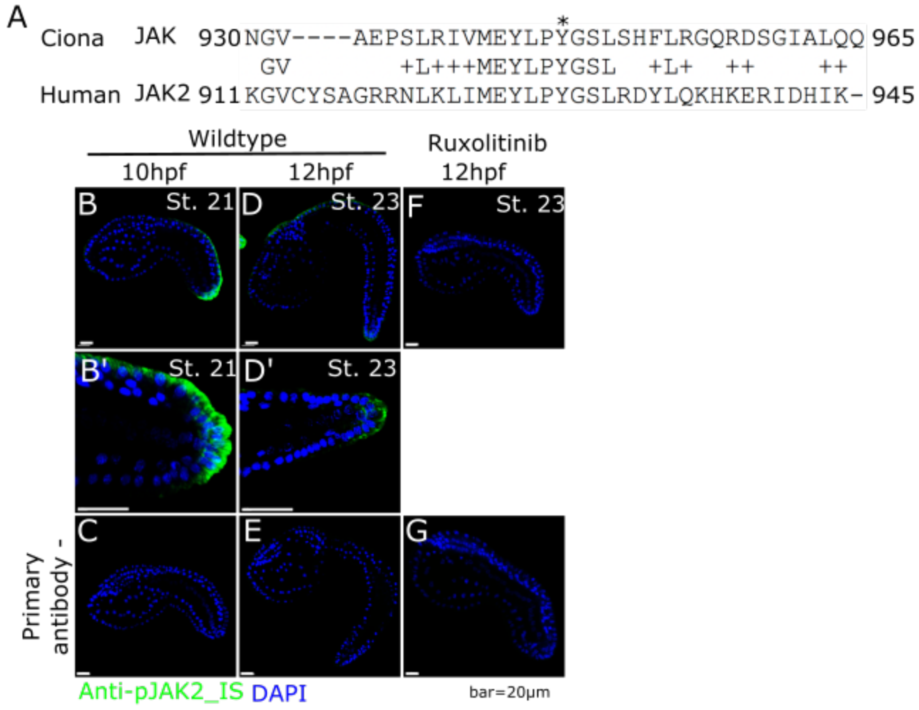

Appendix Figure S5. (A) Aliment of protein sequences of JAK in *Ciona* and JAK2 in Human. Amino acid residues 930-965 in *Ciona* JAK are shown. The conserved actively phosphorylated Tyrosines is marked by *. (B-C) Immunostaining was done with anti-pJAK2 antibody in DMSO control at 10 hpf (B) and its no-primary antibody control (C). The image B is the same image on Figure 3G. (B') Image around the tail tip of B. (D-E) Immunostaining was done with anti-pJAK2 antibody in DMSO control at 12 hpf (D) and its no-primary antibody control (E). (D') Image around the tail tip of D. (F-G) Immunostaining was done with anti-pJAK2 antibody in Ruxolitinib treated embryos at 12 hpf (F) and its no-primary antibody control (G). Scale bars are 20 μm on B-G.

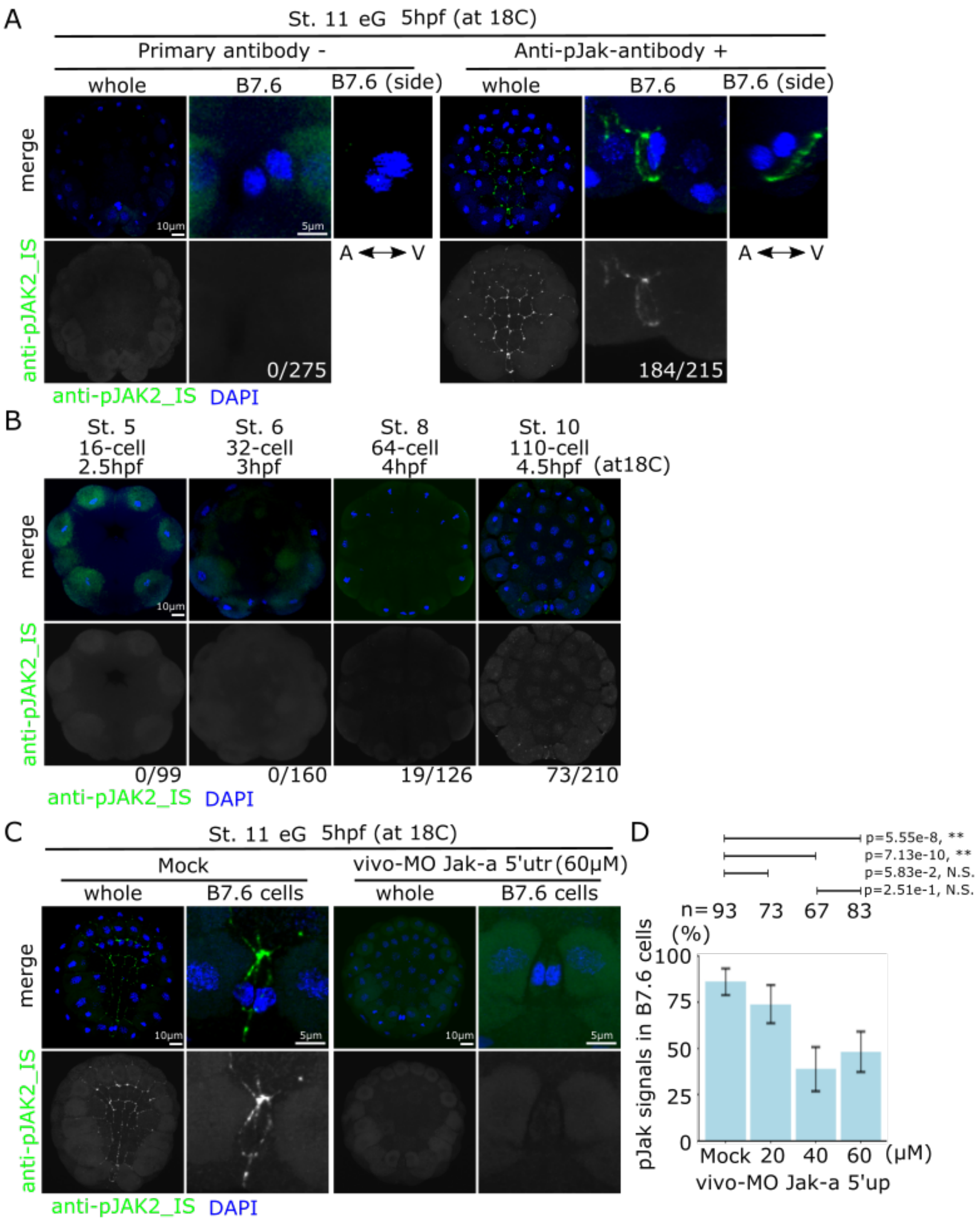

Appendix Figure S6. (A) Immunostaining was done with anti-pJAK2 antibody at early gastrulation (eG) stage with no primary antibody control. The numbers show the embryo showing signals on B7.6 cells out of total embryo. These pictures of whole embryo (whole) and B7.6 cells (B7.6) were taken from vegetal side. The pictures on the side were taken from the side of B7.6 cells. (B) Time series embryos from 16-cell stage to 110-cell stage were stained with anti-pJAK2 antibody immunostaining. These pictures of whole embryo were taken from vegetal side. The numbers show the embryo showing signals on B7.6 cells out of total embryo. (C) The dechorionated eggs were treated in water (Mock) or vivo-MO for *Jak-a* 5' UTR and performed immunostaining with anti-pJAK2 antibody. (D) Rate of embryos showing signals on B7.6 cells. The proportion of the signals in B7.6 cells was shown in y-axis. n means the number of embryos on D. p-value was calculated by t-test on D. p>0.05; N.S, 0.05>p>0.01; *, 0.01>p; **. Nuclei were stained with DAPI showed in blue on A-C. Scale bars are 10μm and 5μm that are indicated in each image on A-C.

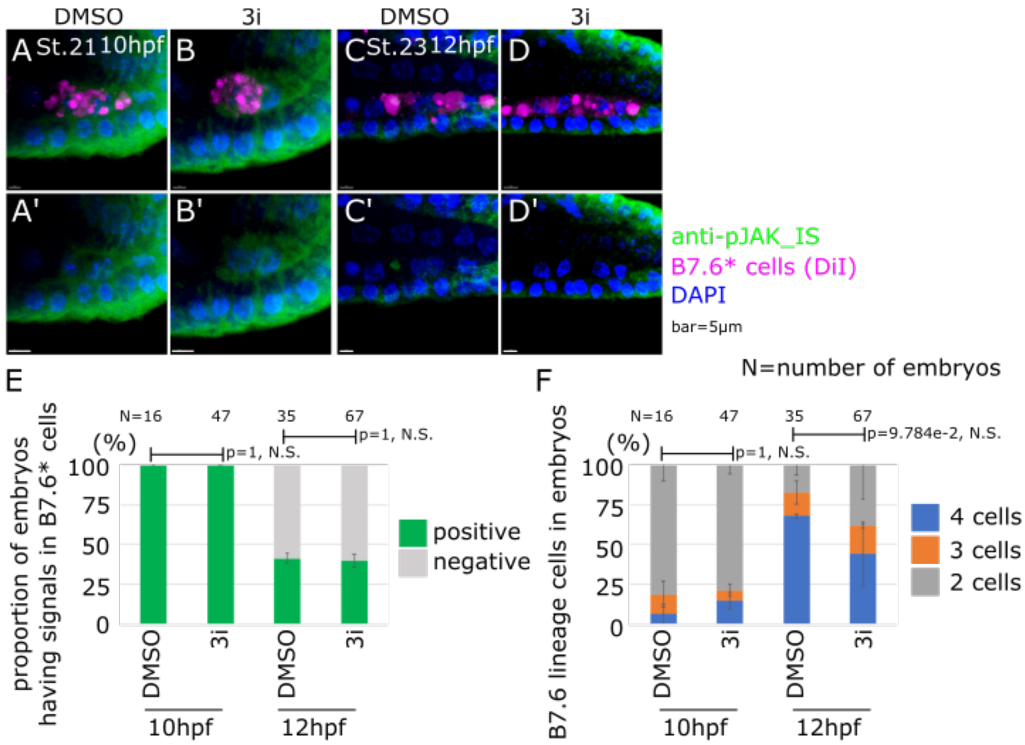

Appendix Figure S7. (A-D) Immunostaining was done with anti-JAK2 antibody under 3i pharmacological inhibitors treatment at 10hpf (A, B) and 12hpf (C, D). Scale bars are 5 μm on A-D. (E) Proportion of signals of immunostaining with anti-pJAK2 antibody in 7.6* cells. The proportion of signal positive nuclei was shown in y-axis. (F) Proportion of cell numbers of B7.6* cells in embryos. The proportion of the number of cells in the embryos was shown in y-axis. Error bars indicate standard error on E and F. p-value was calculated by z-test on E and F. p>0.05; N.S, 0.05>p>0.01; *, 0.01>p; **.

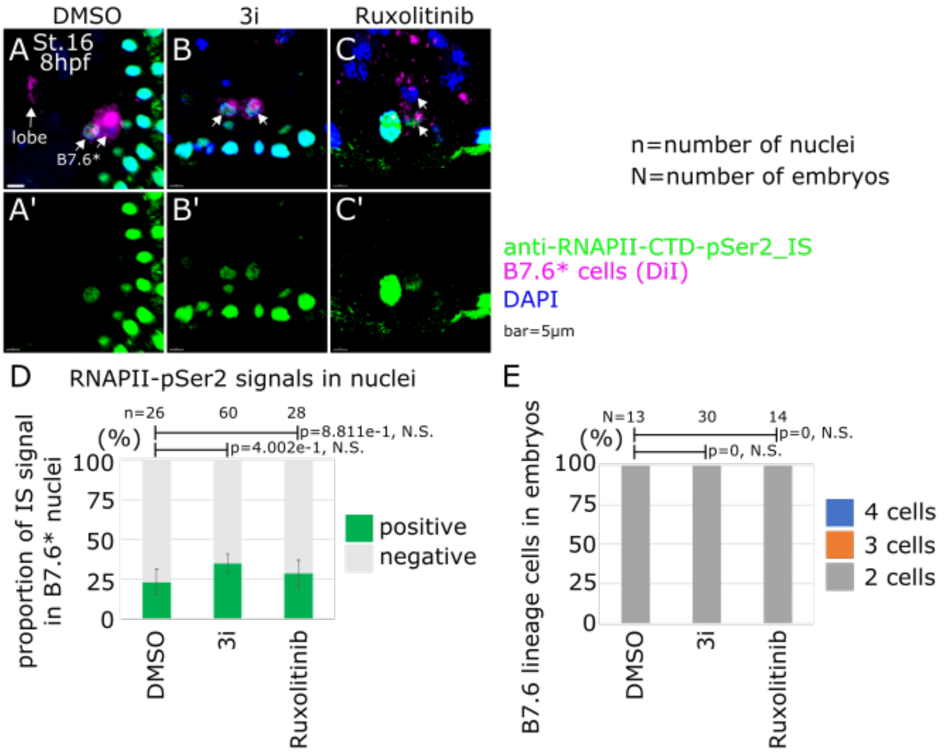

Appendix Figure S8. (A-C) Immunostaining was done with anti-RNAPII-CTD-pSer2 antibody under each pharmacological inhibitor treatment at 8hpf. Scale bar is 5 μm on A. (D) Proportion of signals of immunostaining with anti-RNAPII-CTD-pSer2 antibody in nuclei under each pharmacological inhibitor treatment. The proportion of signal positive nuclei was shown in y-axis. (E) Proportion of cell numbers of B7.6* cells in embryos under each pharmacological inhibitor treatment. The proportion of the number of cells in the embryos was shown in y-axis. Error bars indicate standard error on D and E. p-value was calculated by z-test on D and E. p>0.05; N.S, 0.05>p>0.01; *, 0.01>p; **.

Gene name	Type		Primers sequence
Mef2	intron	Forward	5'-ACTCCATGCCCACTTACGAA-3'
		Reverse	5'-GGATCGCGATGTAAAGGACG-3'
	intron	Forward	5'-CGACACACCCGTTAATCTGG-3'
		Reverse	5'-GGGGATGTAGTCTGAAGGGG-3'
MS4A15/2	exon	Forward	5'-CAAAGGAAATGTCAGTTAC-3'
		Reverse	5'-GTTCCAGAAACTAAATAAG-3'
Pem-1	exon	Forward	5'-AAAGAAAGTGCATAAATATG-3'
		Reverse	5'-AATCGTGAAAAAGTACTAAC-3'

Appendix Table S1. Primers that were used in this paper.

Gene name	KH ID	EST egg	EST tailbud	ISH egg	ISH tail tip
Jak-a	KH.C1.555	+	+	+	+
Jak-b	KH.C8.409	-	-	N.A.	N.A.
Stat-a	KH.C3.827	+	-	+	-
Stat-b	KH.C1.275	+	+	+	+
TGFβ receptor-Ia	KH.S678.1	+	-	+	-
TGFβ receptor-Ib	KH.C14.43	+	-	+	-
TGFβ receptor-Ic	KH.L22.40	+	+	-	+
TGFβ receptor-IIa	KH.C1.598	+	+	+	+
TGFβ receptor-IIb	KH.L141.39	+	-	+	-
Smad1/5/9	KH.C2.573	+	+	+	-
Smad2/3.a	KH.C6.22	+	+	+	-
Smad2/3.b	KH.C12.26	+	-	+	+
FGFR	KH.S742.2	+	+	+	+
Mapk1/2	KH.L153.20	+	+	N.A.	N.A.
Mapk4/6	KH.S606.2	+	+	+	-
Mapk7	KH.C5.69	+	+	+	-
Mapk8/9/10	KH.C14.416	+	+	+	-
MAPKK1/2	KH.L147.22	+	+	+	+
ets/pointed1	KH.C11.10	-	-	-	-
ets/pointed2	KH.C10.113	+	+	+	-
Gsk3	KH.L65.6	+	+	+	+
LRP5/6	KH.L154.1	+	+	+	+
Fz3/6	KH.L9.43	-	+	N.A.	N.A.
Fz4	KH.C6.162	-	-	+	-
Fz5/8	KH.C9.260	-	+	+	-
Orphan Fz-a	KH.C9.236	+	+	+	-
Orphan Fz-b	KH.C12.511	-	+	N.A.	N.A.

Appendix Table S2. Information of genes related to Jak, TGF, FGF and Wnt signaling pathways.
Reference 1; Satou et al., 2005, Zoolog Sci. Aug;22(8):837-843.
Reference 2; Imai et al., 2004, Development. Aug;131(16):4047-58.
