# Supplementary figures and images for: Cellular remodeling and JAK inhibition promote zygotic gene expression in the Ciona germline
Source: EMBO Rep. 2024 Apr 22;25(5):2188–201. doi: 10.1038/s44319-024-00139-0 (PMC11094015; doi:10.1038/s44319-024-00139-0)

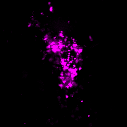

Supplement: Supplementary file 4 — Source data Fig. 1 [file 44319_2024_139_MOESM4_ESM.zip › Figure 1/1MN/DiI_Cyto_Neu_1_Magenta.tif]

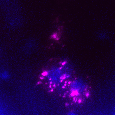

Supplement: Supplementary file 4 — Source data Fig. 1 [file 44319_2024_139_MOESM4_ESM.zip › Figure 1/1MN/S16_DiI_BM.tif]

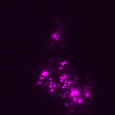

Supplement: Supplementary file 4 — Source data Fig. 1 [file 44319_2024_139_MOESM4_ESM.zip › Figure 1/1MN/S16_DiI_M.tif]

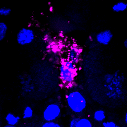

Supplement: Supplementary file 4 — Source data Fig. 1 [file 44319_2024_139_MOESM4_ESM.zip › Figure 1/1MN/DiI_Cyto_Neu_1_BlueMagenta.tif]

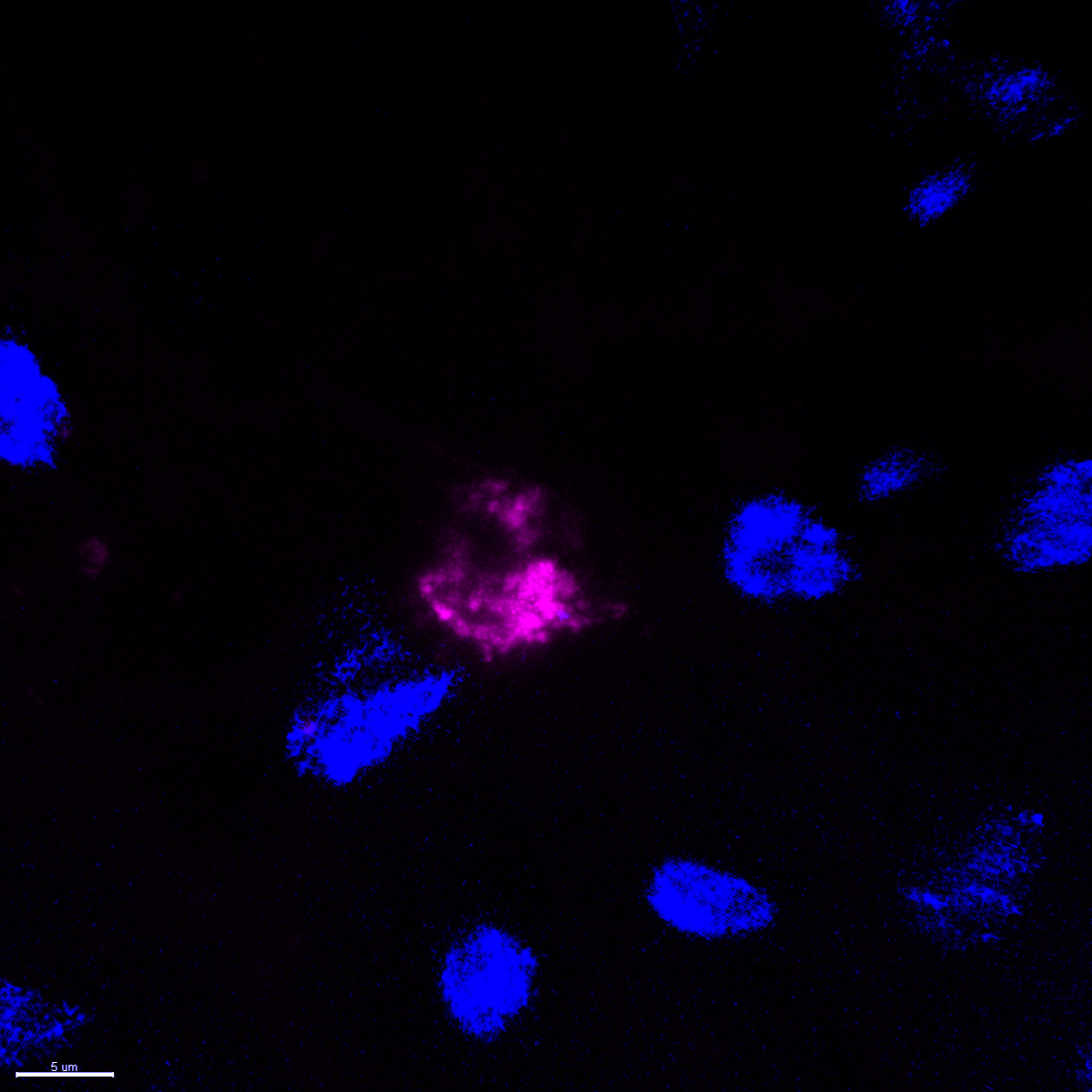

Supplement: Supplementary file 4 — Source data Fig. 1 [file 44319_2024_139_MOESM4_ESM.zip › Figure 1/1H/lobe.tif]

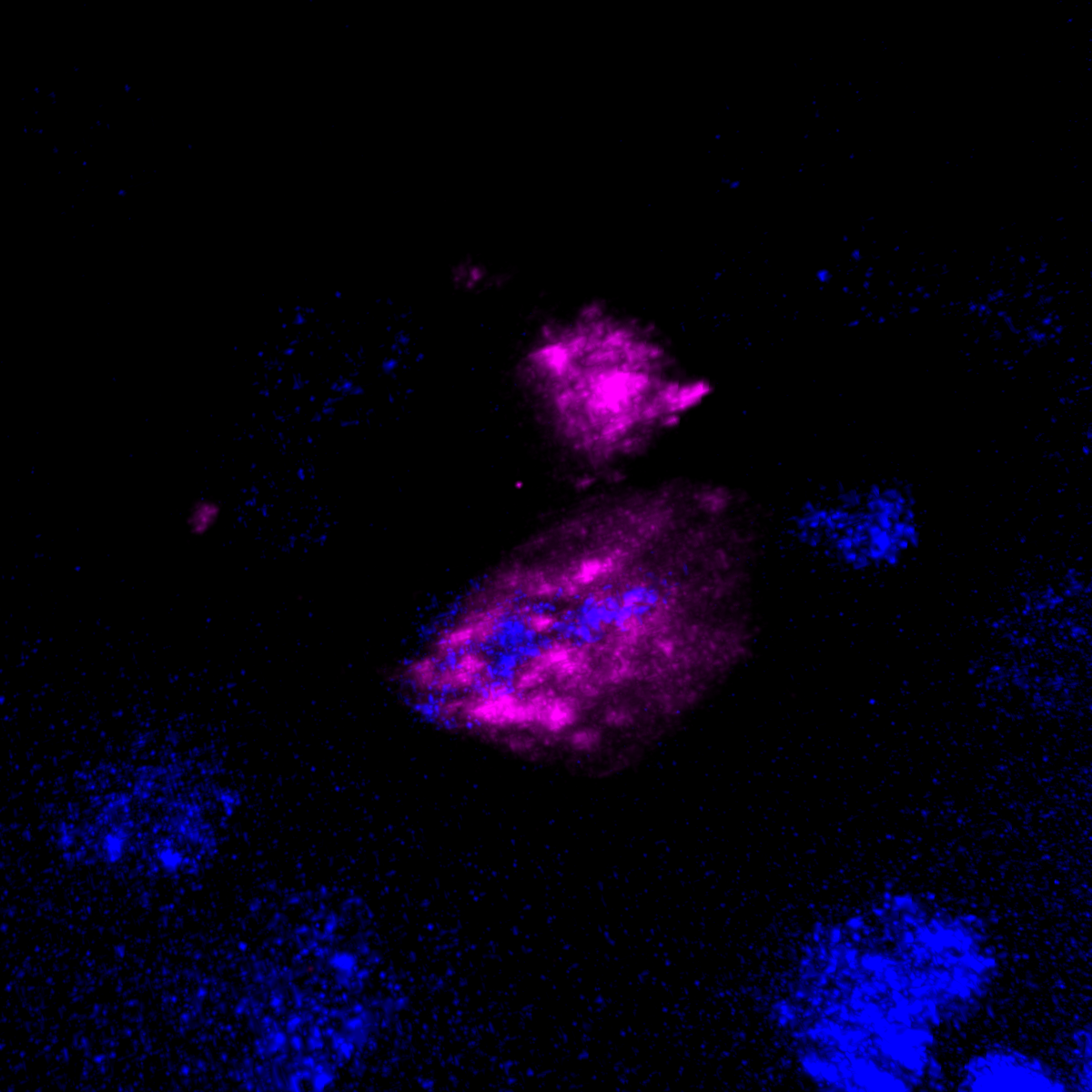

Supplement: Supplementary file 4 — Source data Fig. 1 [file 44319_2024_139_MOESM4_ESM.zip › Figure 1/1F/lN_lobescission_DR.tif]

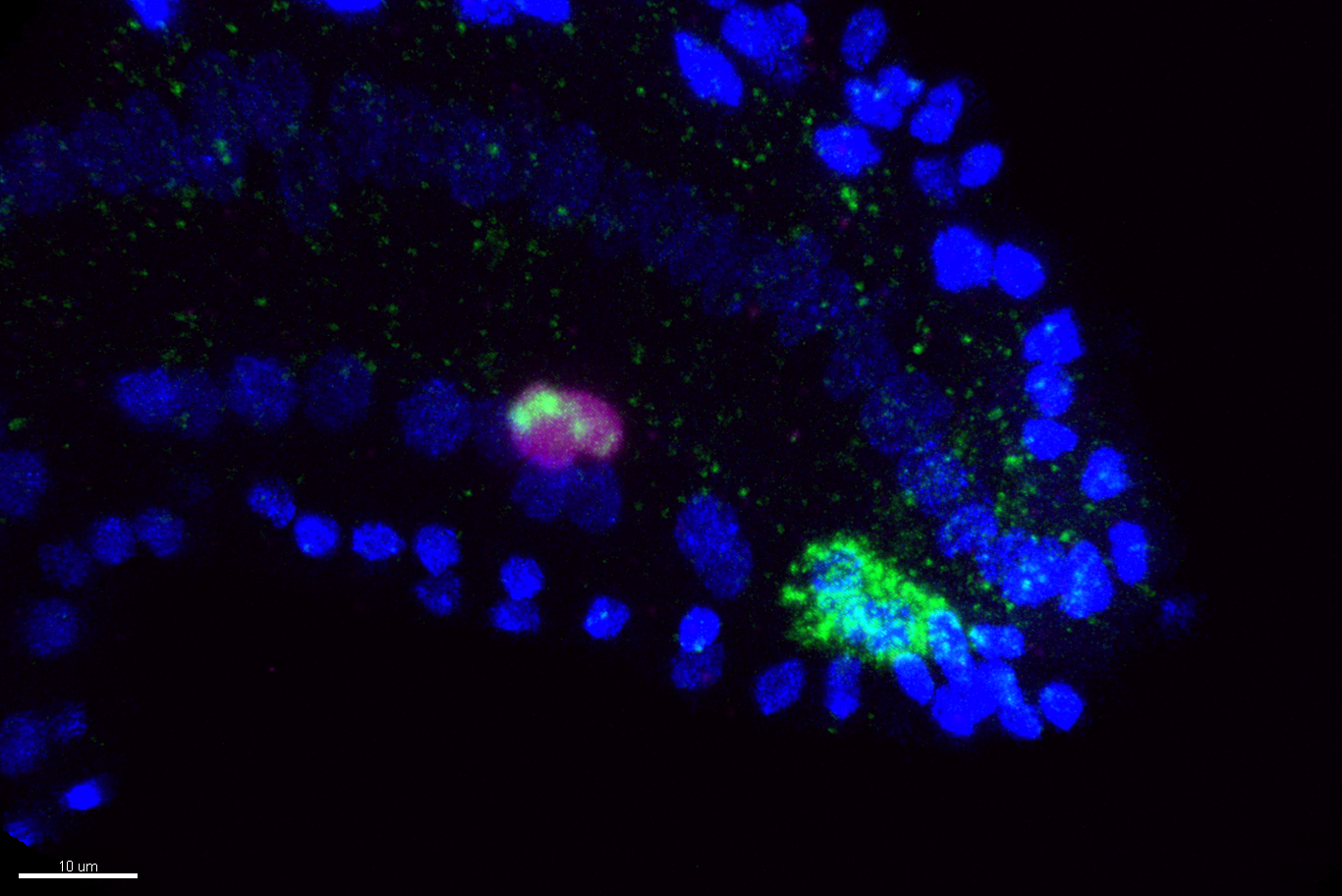

Supplement: Supplementary file 4 — Source data Fig. 1 [file 44319_2024_139_MOESM4_ESM.zip › Figure 1/1A/c24_G_pem_R.tif]

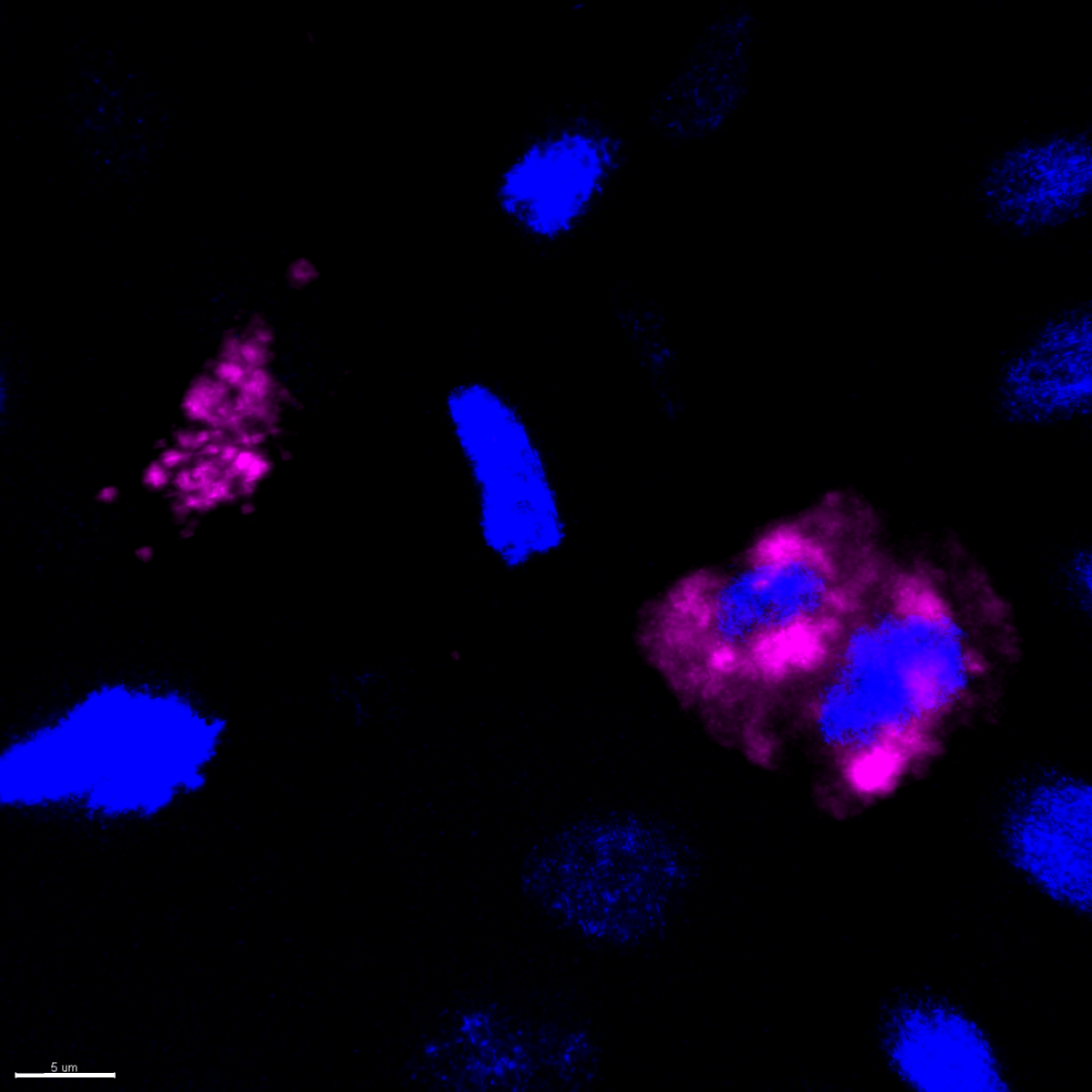

Supplement: Supplementary file 4 — Source data Fig. 1 [file 44319_2024_139_MOESM4_ESM.zip › Figure 1/1G/lN_lobeScission_separate_DR.tif]

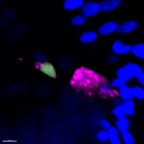

Supplement: Supplementary file 4 — Source data Fig. 1 [file 44319_2024_139_MOESM4_ESM.zip › Figure 1/1L/Dii_pem1_ISH_lN_lat.jpg]

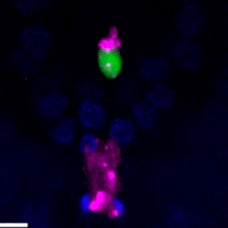

Supplement: Supplementary file 4 — Source data Fig. 1 [file 44319_2024_139_MOESM4_ESM.zip › Figure 1/1K/Dii_Pem1_ISH_lN_veg.jpg]

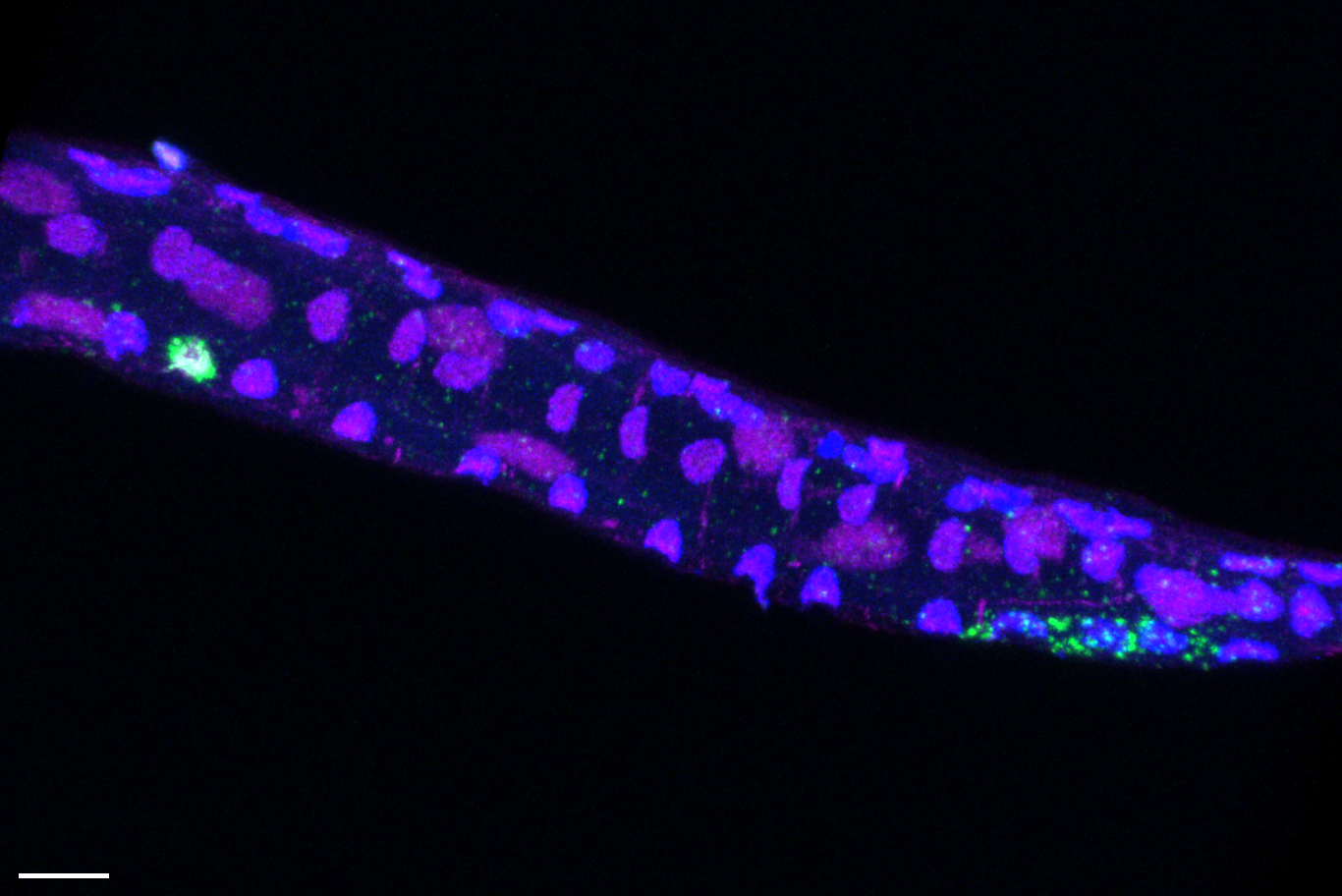

Supplement: Supplementary file 4 — Source data Fig. 1 [file 44319_2024_139_MOESM4_ESM.zip › Figure 1/1B/c24_pSer5_12hpf_DGR.tif]

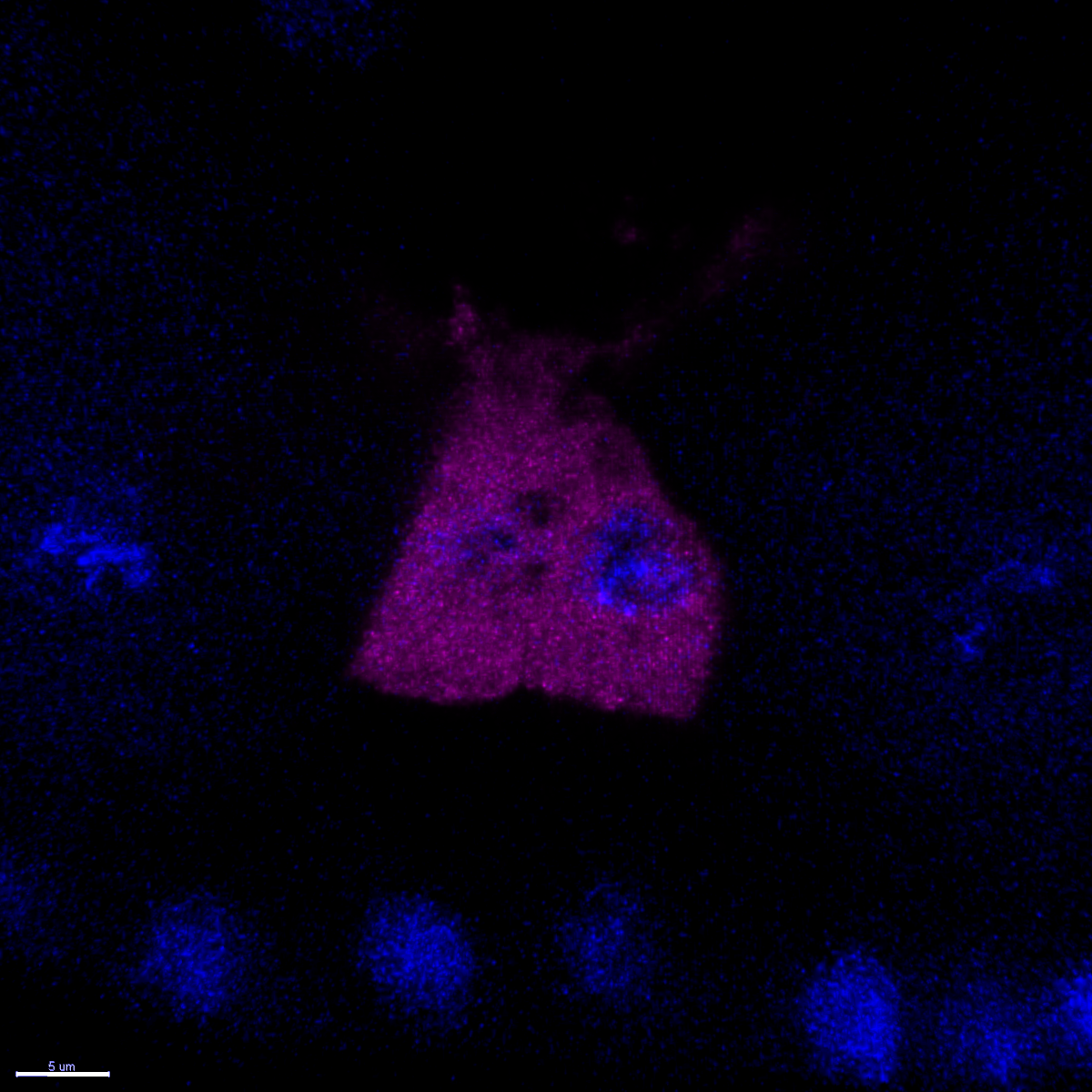

Supplement: Supplementary file 4 — Source data Fig. 1 [file 44319_2024_139_MOESM4_ESM.zip › Figure 1/1E/eG_DiI_B7.6_ventral.tif]

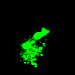

Supplement: Supplementary file 4 — Source data Fig. 1 [file 44319_2024_139_MOESM4_ESM.zip › Figure 1/1P/DiICyto_PemISH_G.tif]

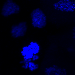

Supplement: Supplementary file 4 — Source data Fig. 1 [file 44319_2024_139_MOESM4_ESM.zip › Figure 1/1P/DiICyto_pemISH_B.tif]

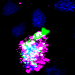

Supplement: Supplementary file 4 — Source data Fig. 1 [file 44319_2024_139_MOESM4_ESM.zip › Figure 1/1P/DiICyto_pemISH_GBM.tif]

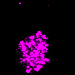

Supplement: Supplementary file 4 — Source data Fig. 1 [file 44319_2024_139_MOESM4_ESM.zip › Figure 1/1P/DiICyto_pemISH_M.tif]

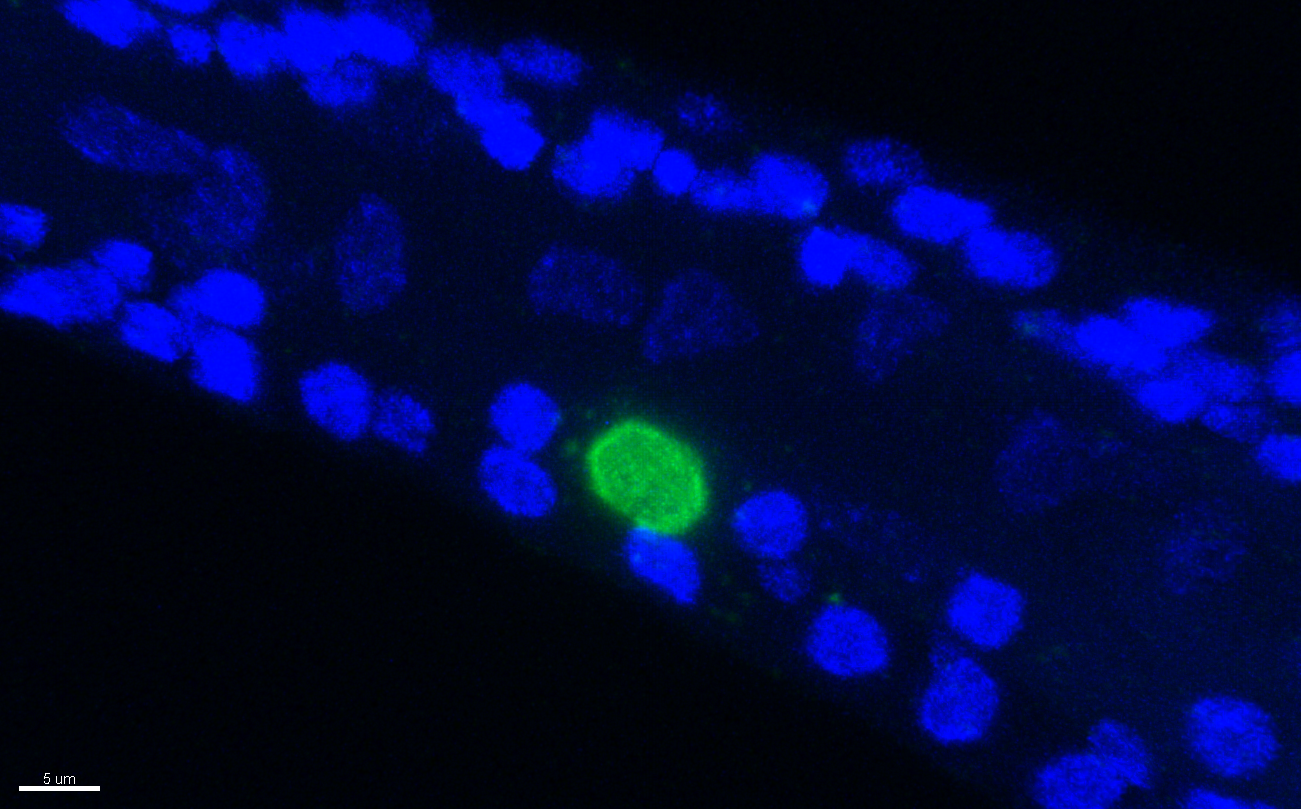

Supplement: Supplementary file 4 — Source data Fig. 1 [file 44319_2024_139_MOESM4_ESM.zip › Figure 1/1C/pem-1_pSer5_16hpf_DG.tif]

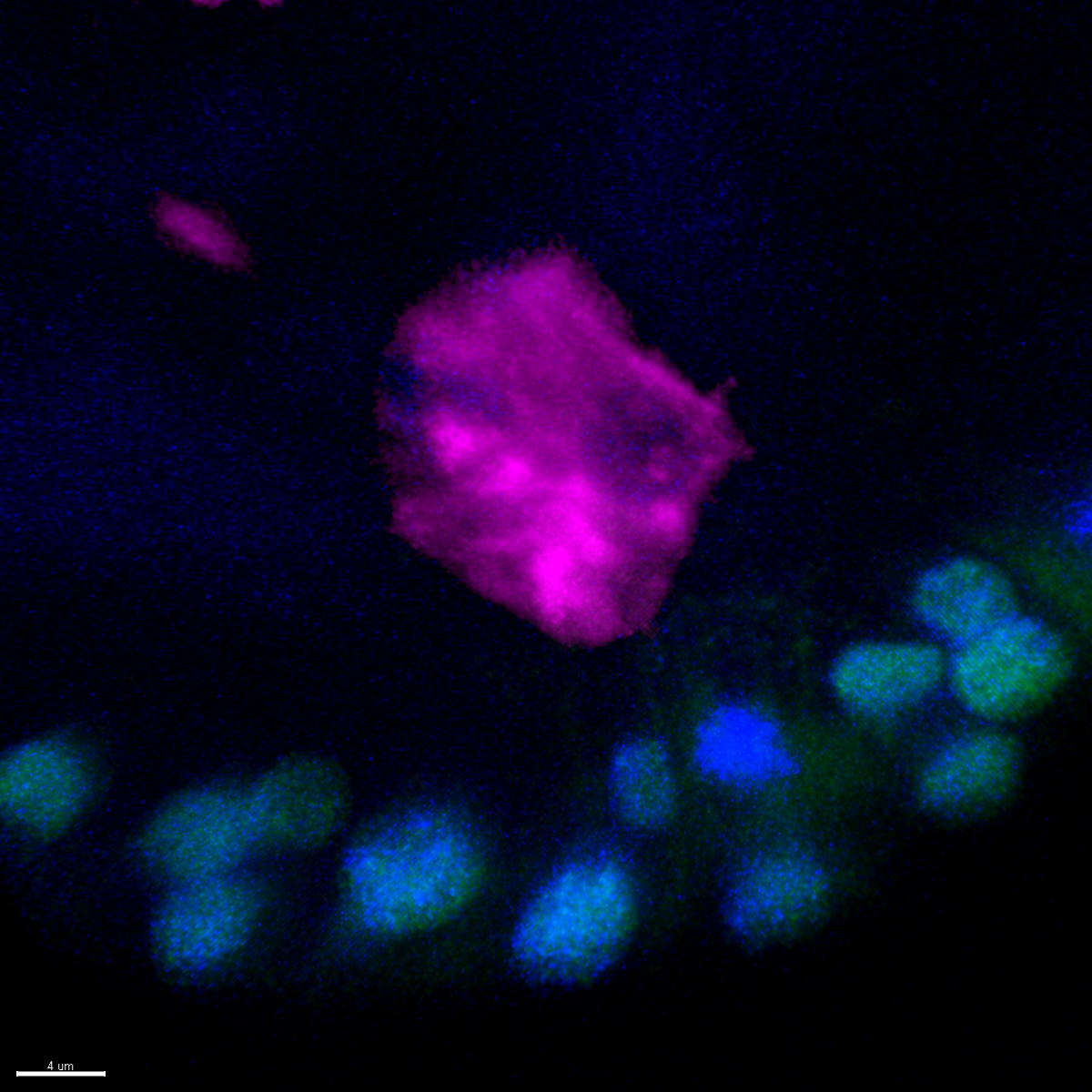

Supplement: Supplementary file 5 — Source data Fig. 2 [file 44319_2024_139_MOESM5_ESM.zip › Figure 2/2A/071820_DiI_pSer2_[ii4_8hpf_2_Image_5]_2020-07-25T10-53-11.590.tif]

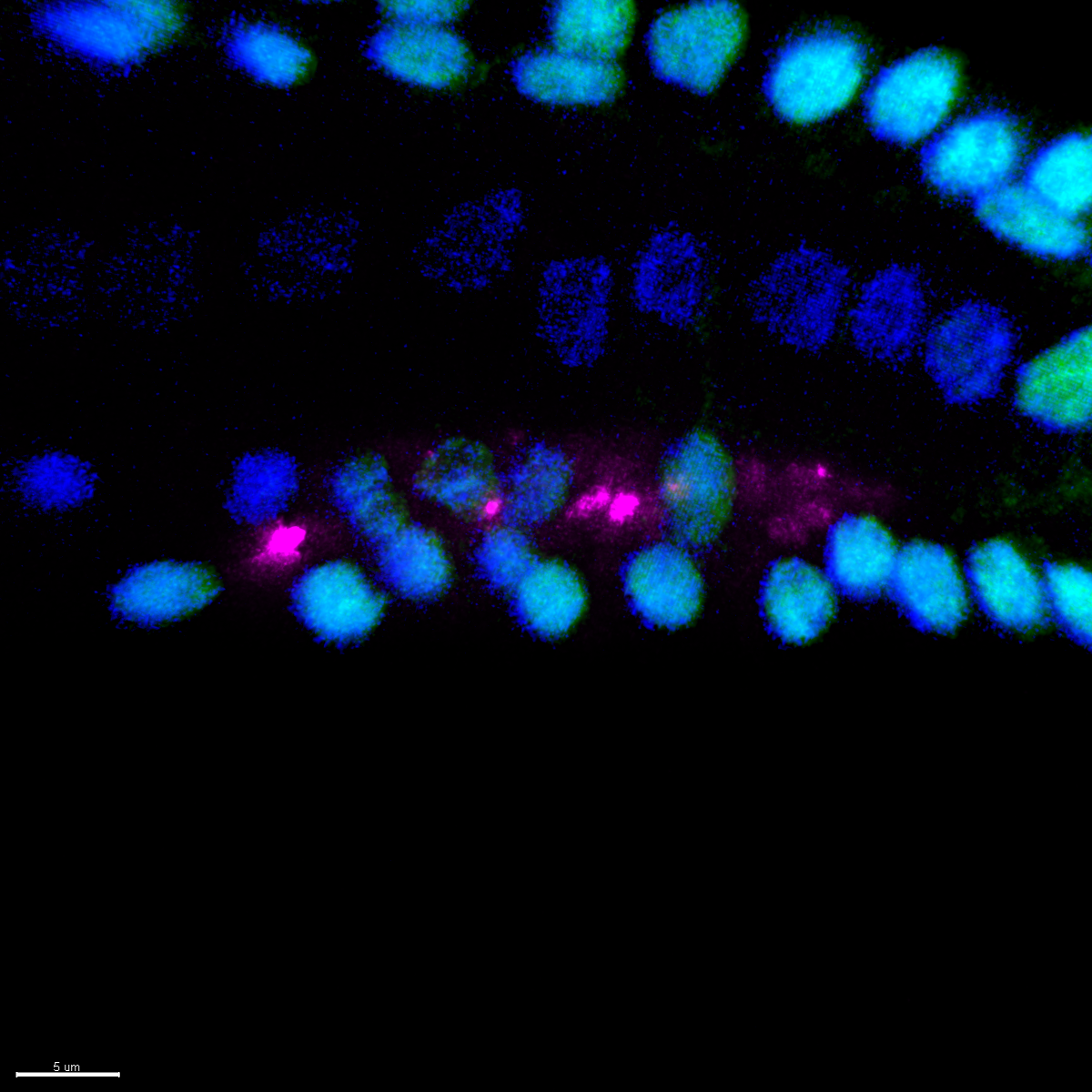

Supplement: Supplementary file 5 — Source data Fig. 2 [file 44319_2024_139_MOESM5_ESM.zip › Figure 2/2C/071820_DiI_pSer2_[ii16_12hpf_2_Image_17]_2020-07-25T11-08-48.963.tif]

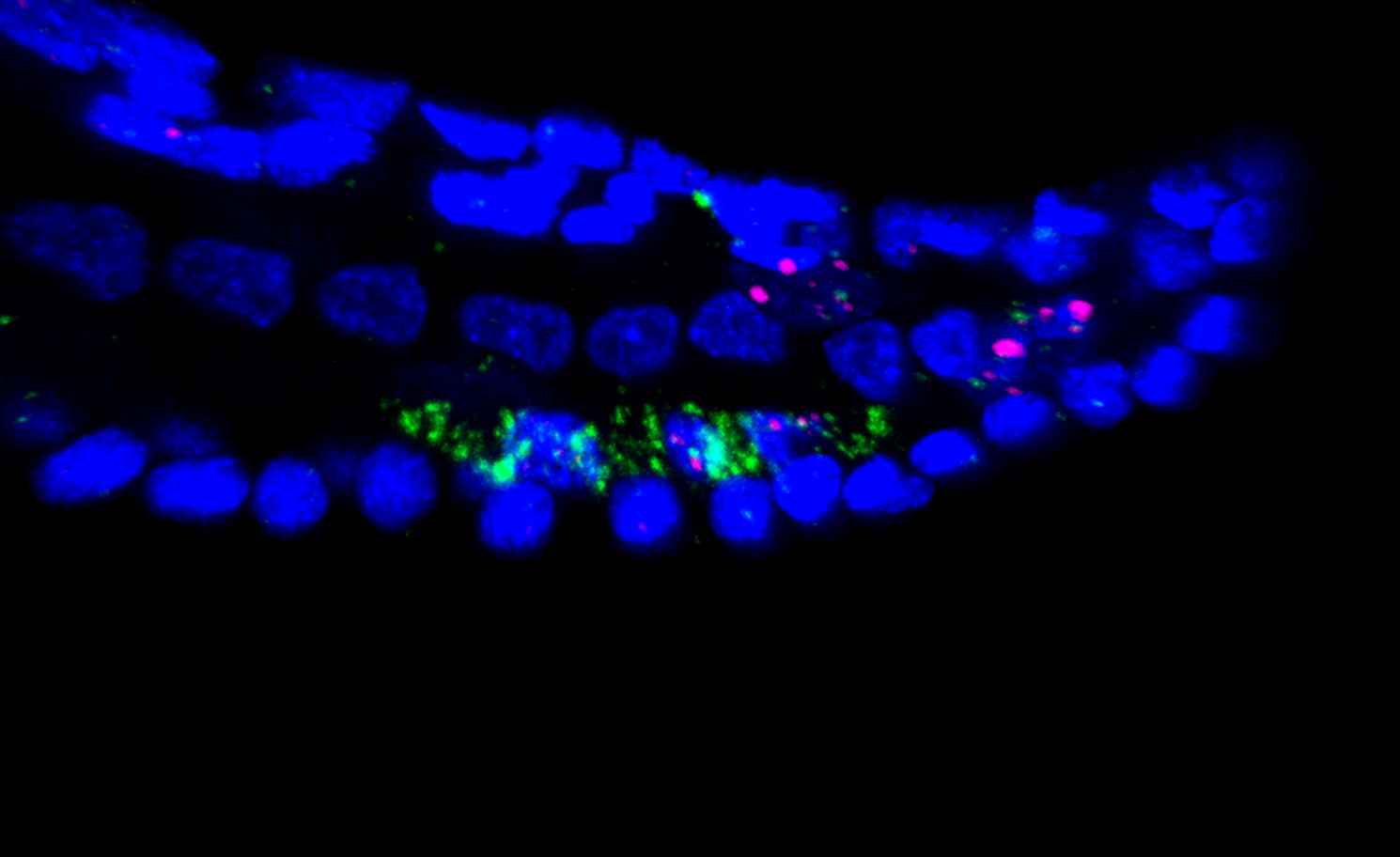

Supplement: Supplementary file 5 — Source data Fig. 2 [file 44319_2024_139_MOESM5_ESM.zip › Figure 2/2E/Mef23_c24_12hpf_DGR.tif]

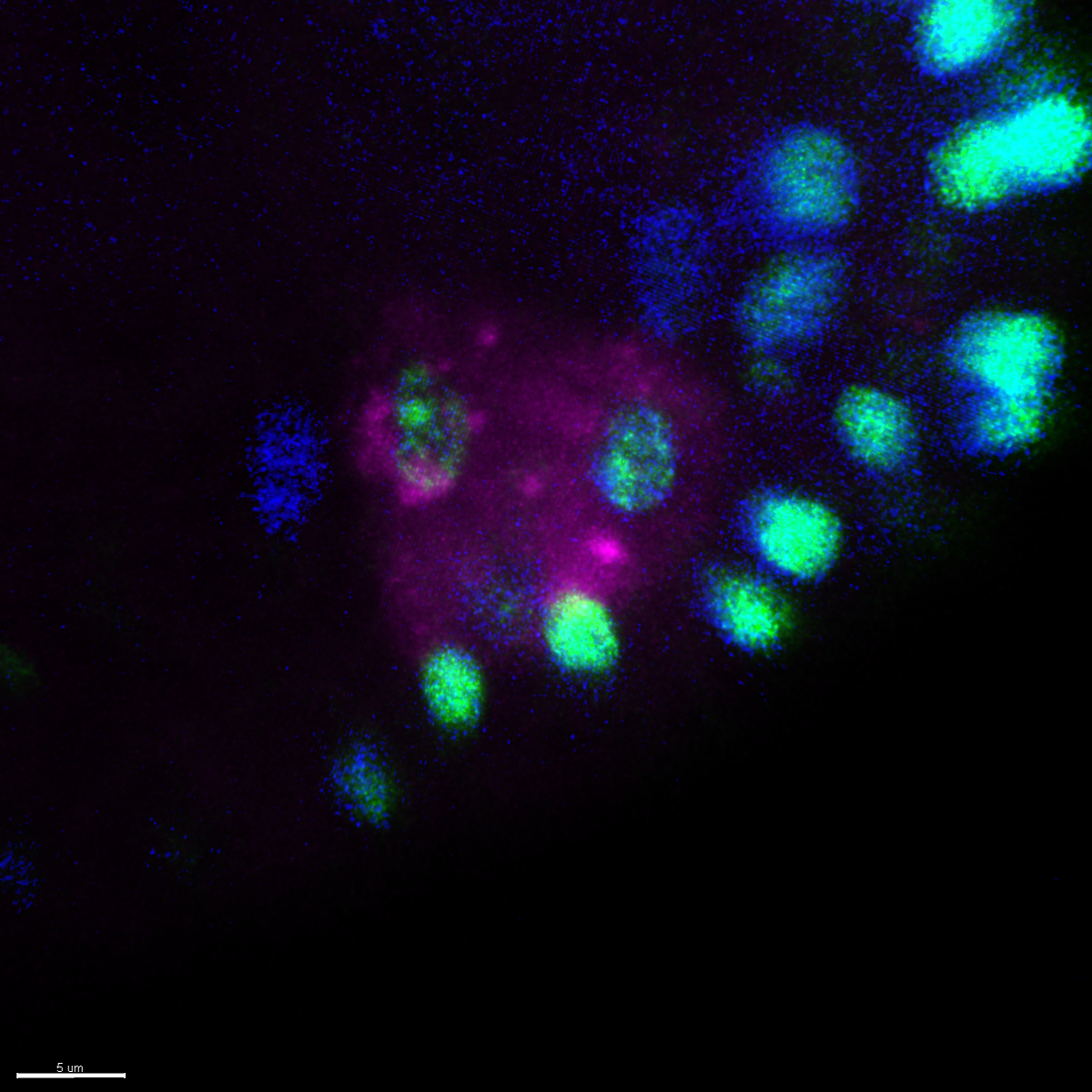

Supplement: Supplementary file 5 — Source data Fig. 2 [file 44319_2024_139_MOESM5_ESM.zip › Figure 2/2B/071820_DiI_pSer2_[ii12_10hph_6_posi_Image_13]_2020-07-25T11-02-08.866.tif]

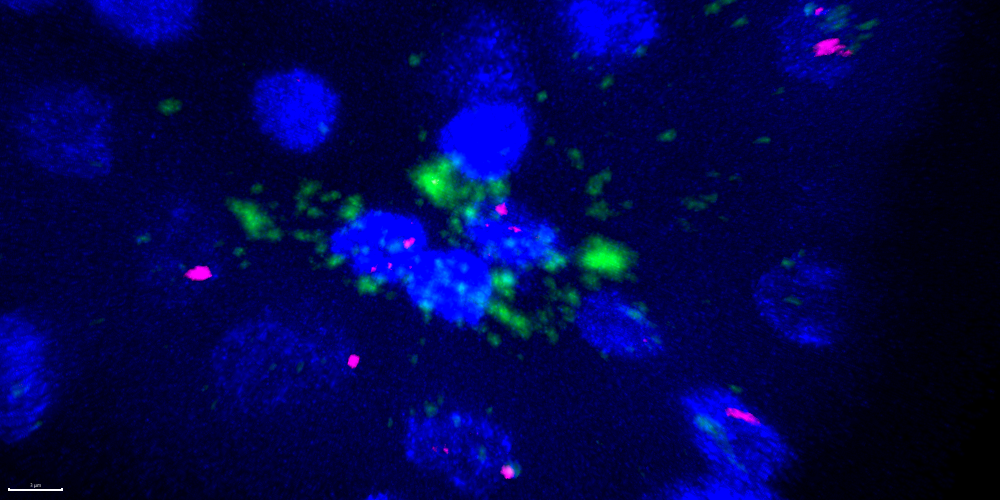

Supplement: Supplementary file 6 — Source data Fig. 3 [file 44319_2024_139_MOESM6_ESM.zip › Figure 3/3L/012321_Mef2_8-12_JAKinh_[ii2_1_DMSO_8-10hpf_3_Image_3]_2021-01-25T15-00-10.939.tif]

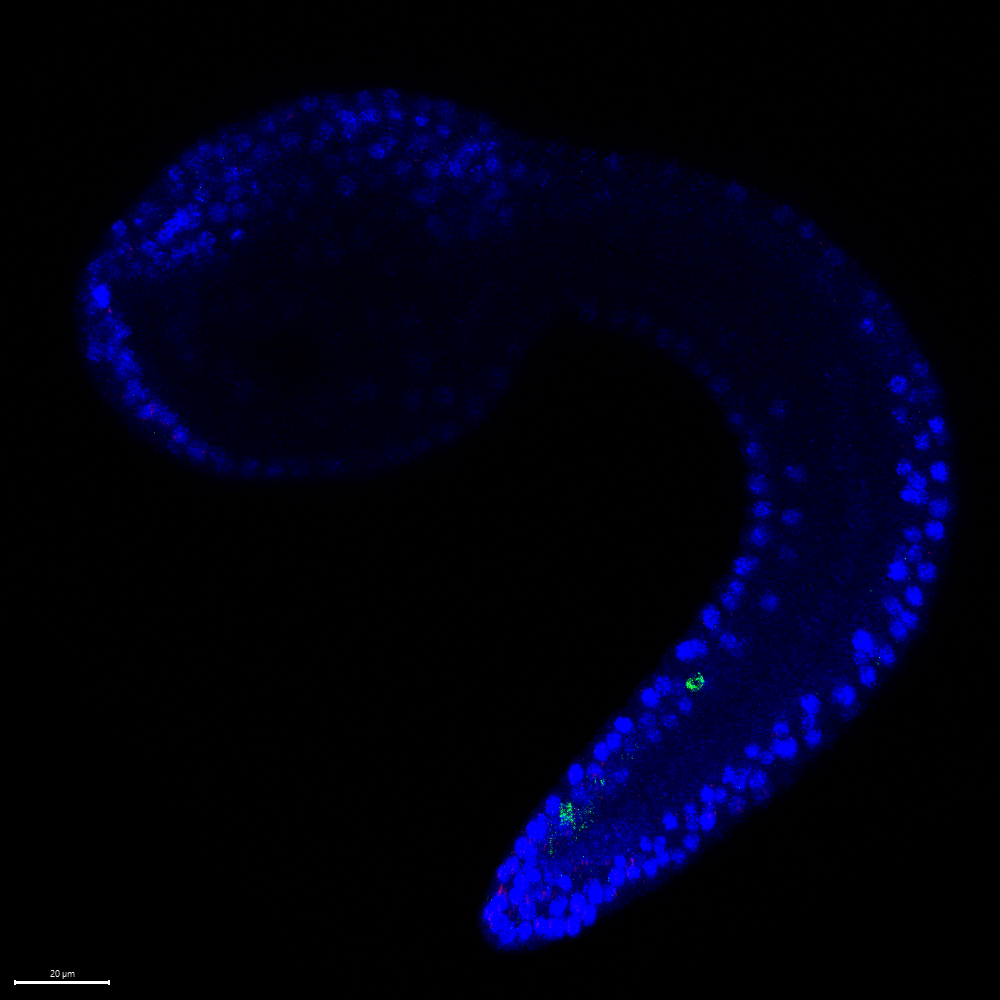

Supplement: Supplementary file 6 — Source data Fig. 3 [file 44319_2024_139_MOESM6_ESM.zip › Figure 3/3B/012221_Mef2_3i_3iR_[ii0_6_3i_1_whole_Image_1]_2021-01-25T14-33-43.746.tif]

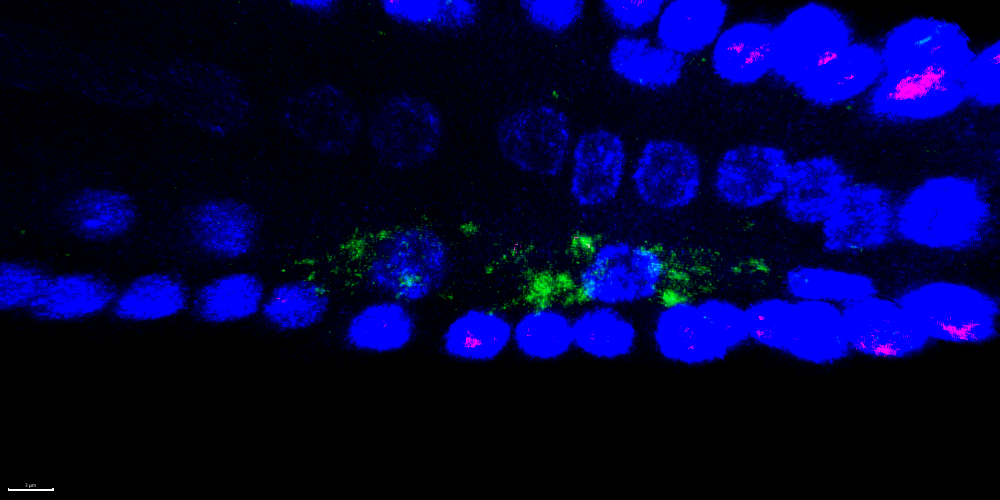

Supplement: Supplementary file 6 — Source data Fig. 3 [file 44319_2024_139_MOESM6_ESM.zip › Figure 3/3B/012221_Mef2_3i_3iR_[ii2_6_3i_3_Image_3]_2021-01-25T14-45-16.949.tif]

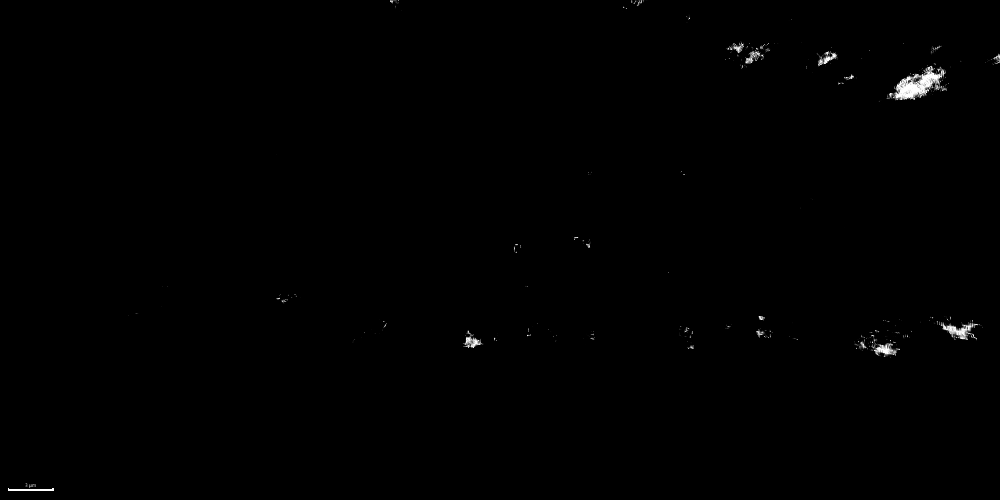

Supplement: Supplementary file 6 — Source data Fig. 3 [file 44319_2024_139_MOESM6_ESM.zip › Figure 3/3B/012221_Mef2_3i_3iR_[ii2_6_3i_3_Image_3]_2021-01-25T14-45-16.949.tif (red).tif]

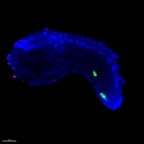

Supplement: Supplementary file 6 — Source data Fig. 3 [file 44319_2024_139_MOESM6_ESM.zip › Figure 3/3C/Mef2_Rux_12hpf_whole.jpg]

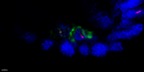

Supplement: Supplementary file 6 — Source data Fig. 3 [file 44319_2024_139_MOESM6_ESM.zip › Figure 3/3C/Mef2_Rux-12hpf_PGCs.jpg]

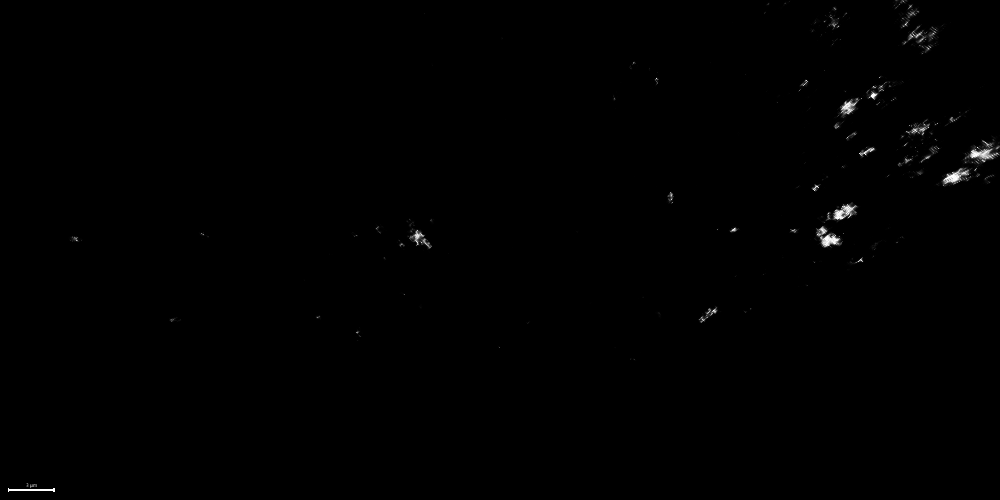

Supplement: Supplementary file 6 — Source data Fig. 3 [file 44319_2024_139_MOESM6_ESM.zip › Figure 3/3D/012221_Mef2_3i_3iR_[ii5_7_3iR_2_Image_6]_2021-01-25T14-47-04.460.tif (red).tif]

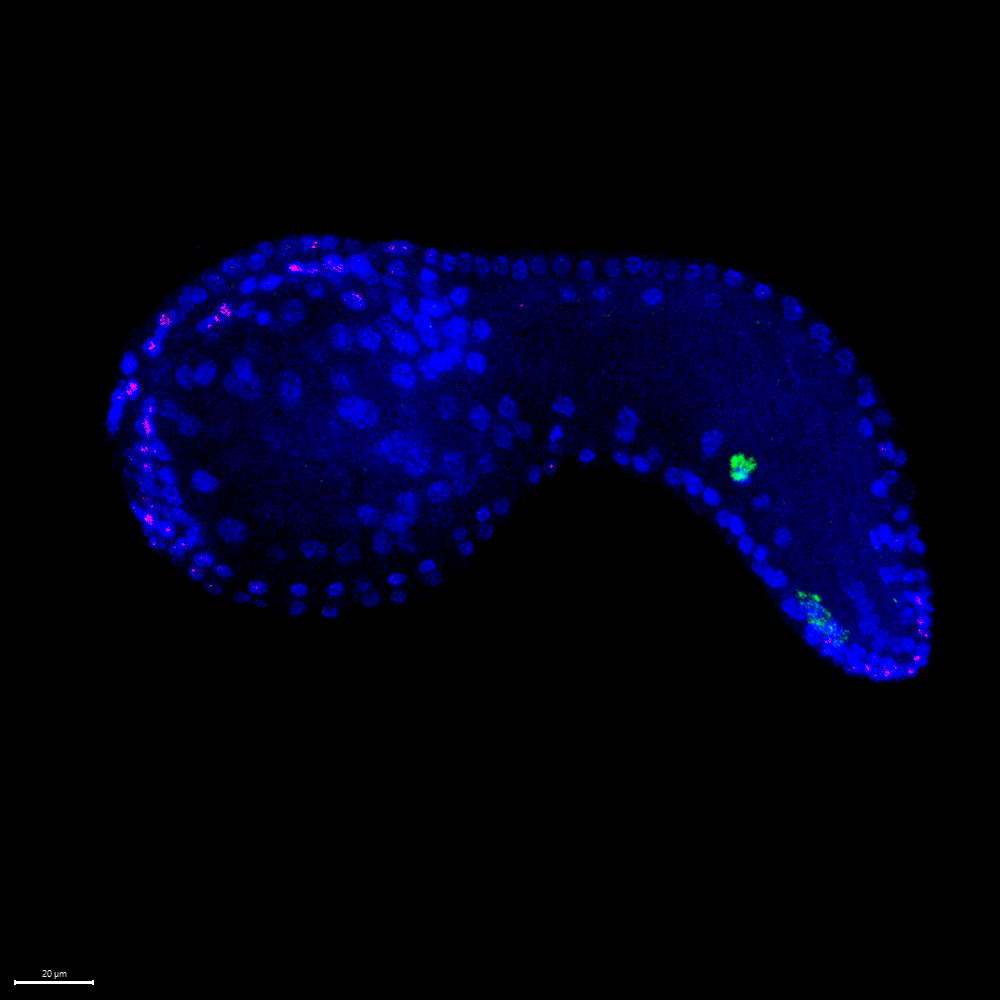

Supplement: Supplementary file 6 — Source data Fig. 3 [file 44319_2024_139_MOESM6_ESM.zip › Figure 3/3D/012221_Mef2_3i_3iR_[ii8_7_3iR_5_whole_Image_9]_2021-01-25T14-36-07.350.tif]

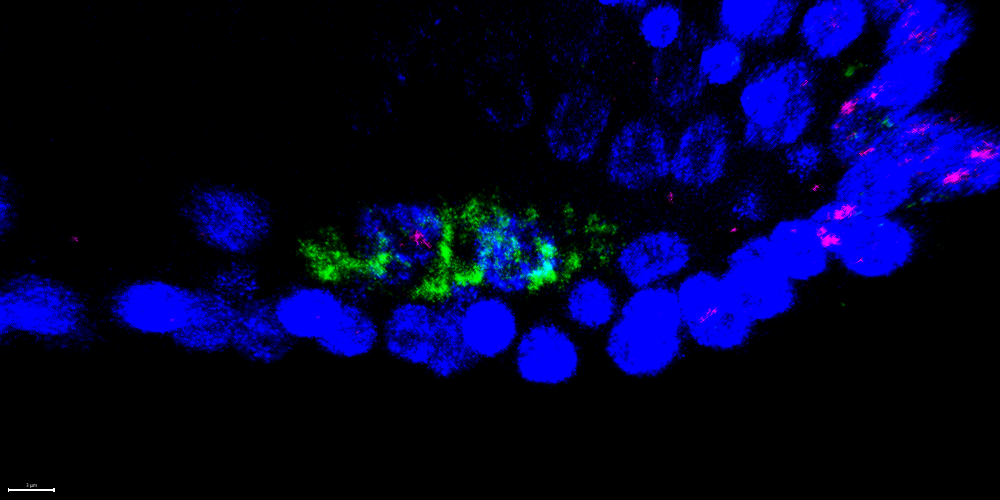

Supplement: Supplementary file 6 — Source data Fig. 3 [file 44319_2024_139_MOESM6_ESM.zip › Figure 3/3D/012221_Mef2_3i_3iR_[ii5_7_3iR_2_Image_6]_2021-01-25T14-47-04.460.tif]

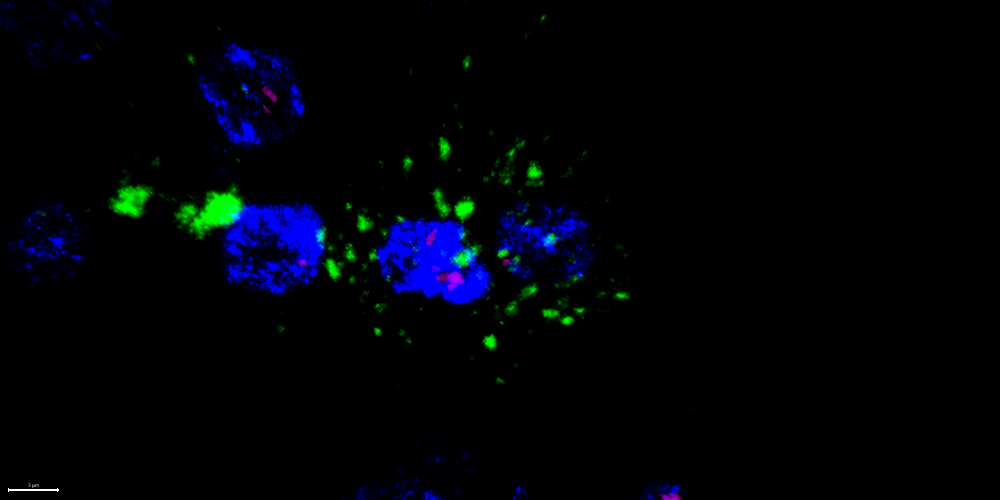

Supplement: Supplementary file 6 — Source data Fig. 3 [file 44319_2024_139_MOESM6_ESM.zip › Figure 3/3M/012321_Mef2_8-12_JAKinh_[ii6_3_Ruxo_8-10hpf_1_Image_7]_2021-01-25T15-04-21.433.tif]

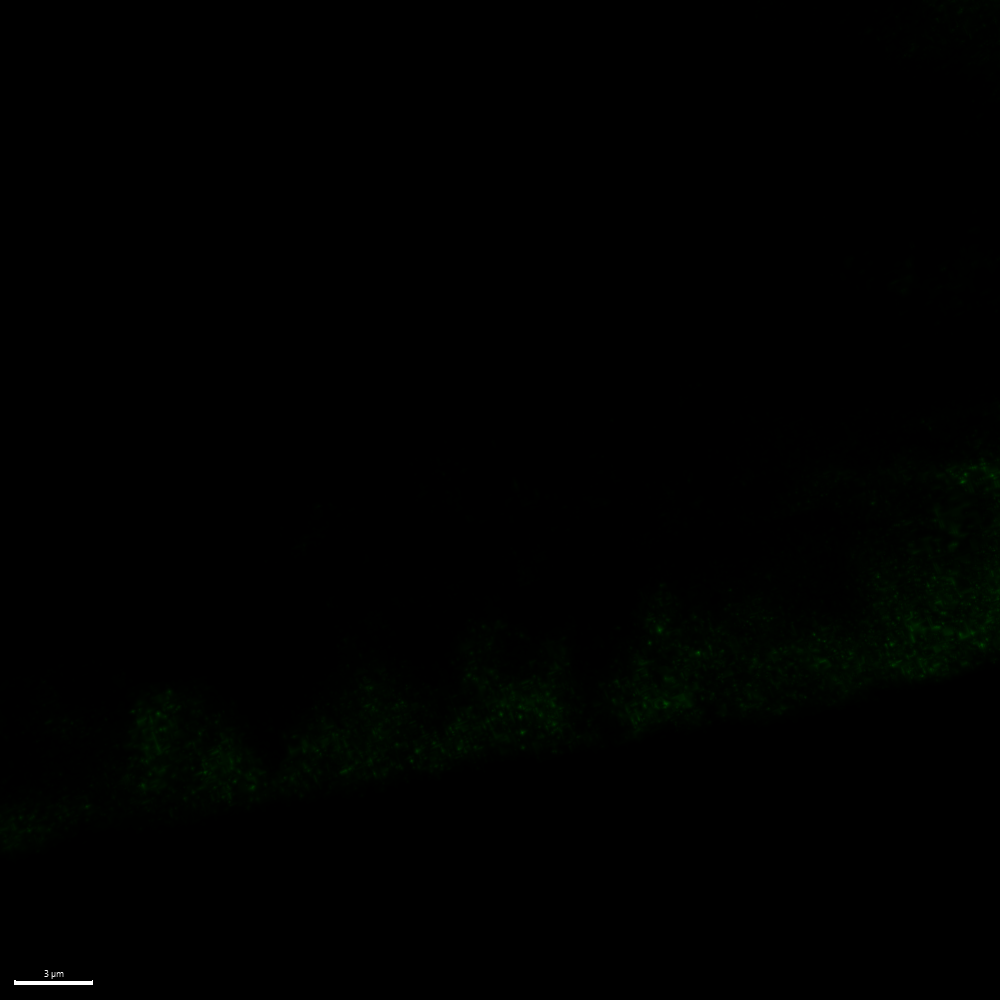

Supplement: Supplementary file 6 — Source data Fig. 3 [file 44319_2024_139_MOESM6_ESM.zip › Figure 3/3J/122020Dii_pJAK_IS_[ii22_2_WT_12hpf_1_Image_23]_2020-12-21T13-38-03.255.tif]

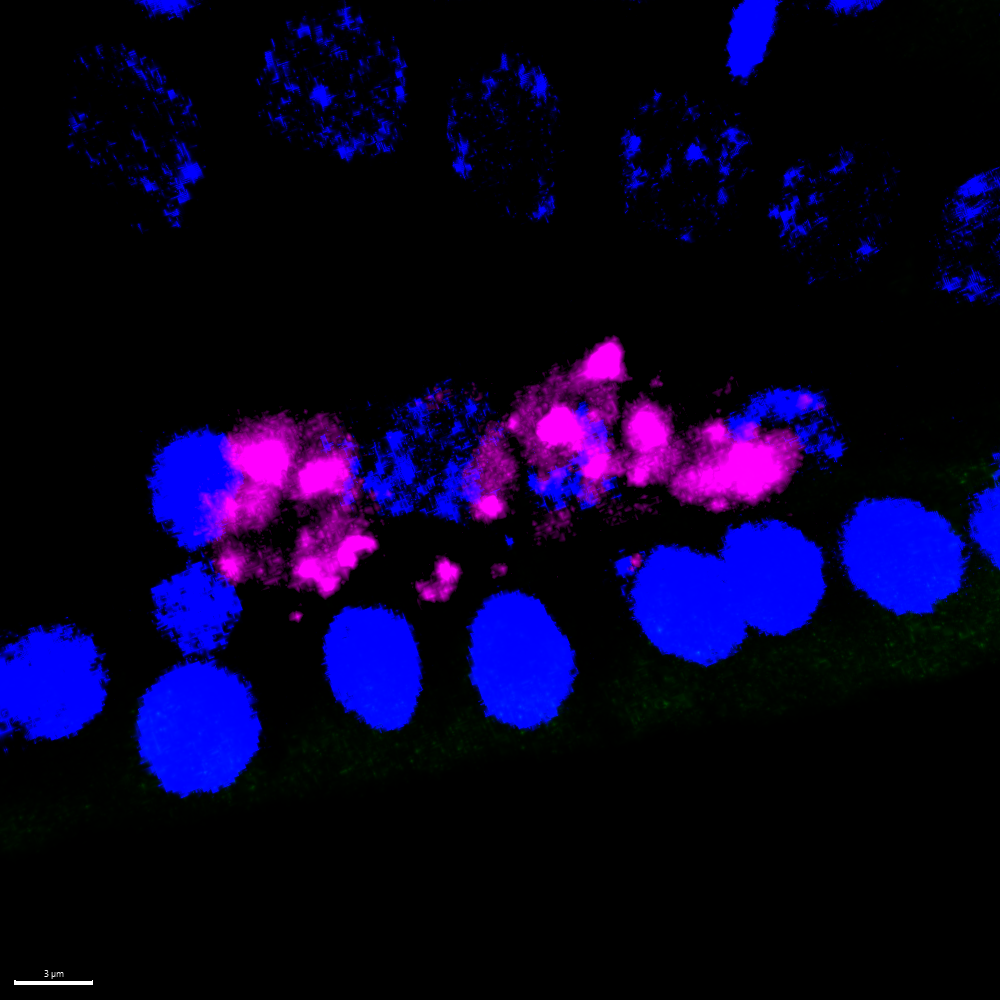

Supplement: Supplementary file 6 — Source data Fig. 3 [file 44319_2024_139_MOESM6_ESM.zip › Figure 3/3J/122020Dii_pJAK_IS_[ii22_2_WT_12hpf_1_Image_23]_2020-12-21T13-37-48.613.tif]

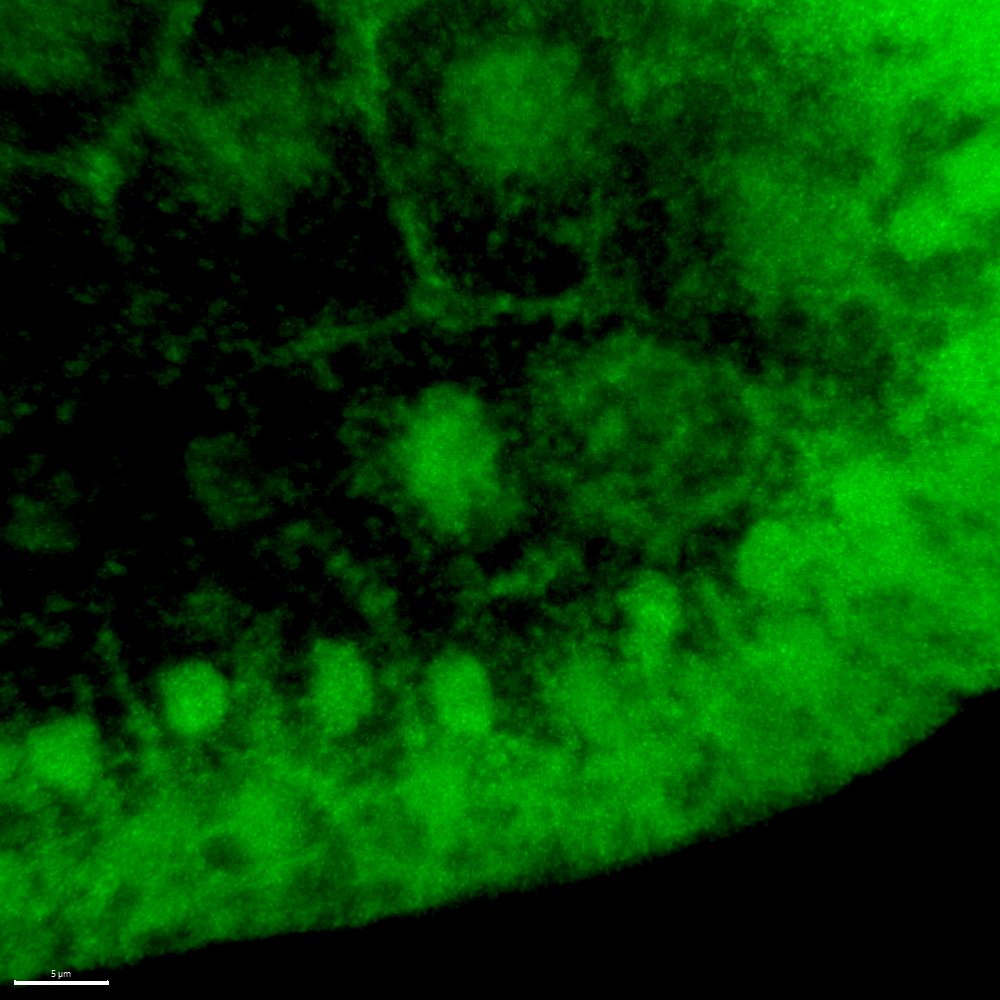

Supplement: Supplementary file 6 — Source data Fig. 3 [file 44319_2024_139_MOESM6_ESM.zip › Figure 3/3H/122020Dii_pJAK_IS_[ii6_1_WT_10hpf_7_Image_7]_2020-12-21T13-32-43.333.jpg]

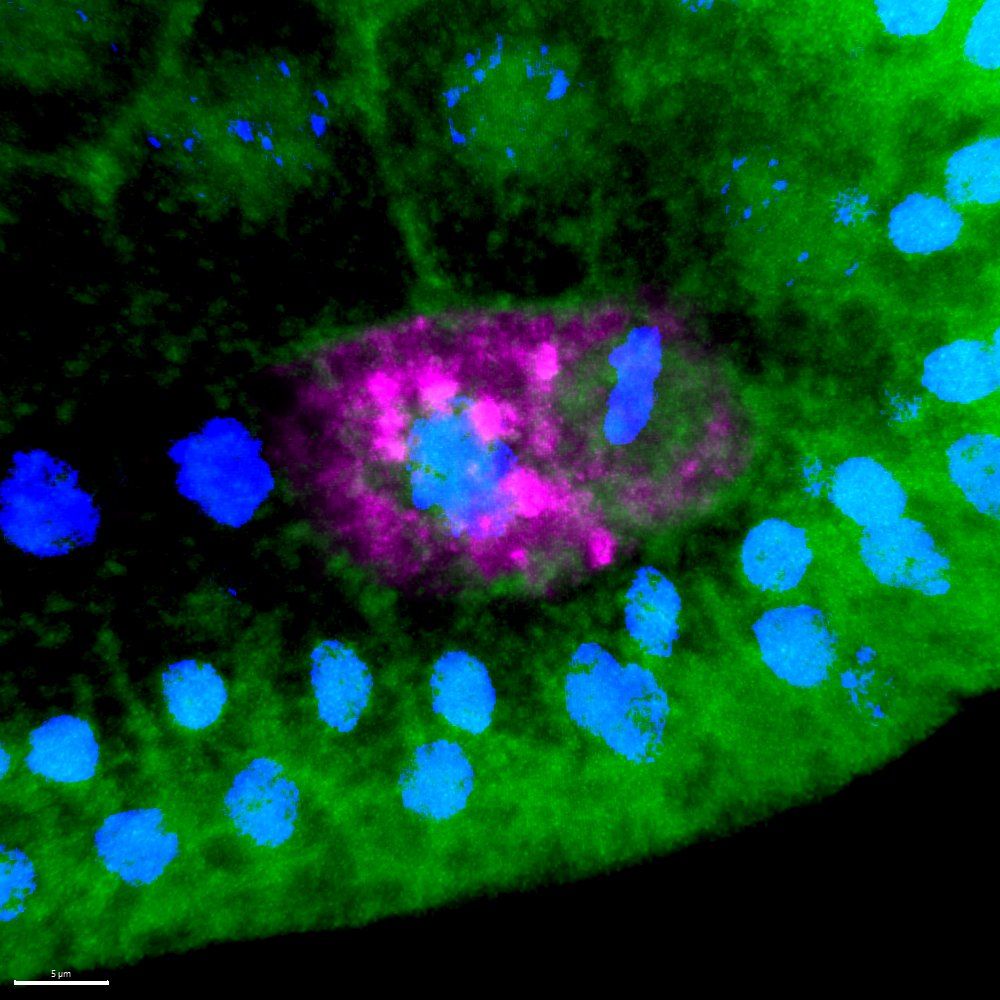

Supplement: Supplementary file 6 — Source data Fig. 3 [file 44319_2024_139_MOESM6_ESM.zip › Figure 3/3H/122020Dii_pJAK_IS_[ii6_1_WT_10hpf_7_Image_7]_2020-12-21T13-32-34.930.jpg]

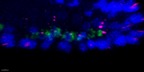

Supplement: Supplementary file 6 — Source data Fig. 3 [file 44319_2024_139_MOESM6_ESM.zip › Figure 3/3A/Mef2_DMSO_12hpf_PGCs.jpg]

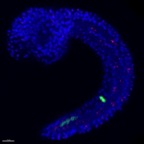

Supplement: Supplementary file 6 — Source data Fig. 3 [file 44319_2024_139_MOESM6_ESM.zip › Figure 3/3A/Mef2_DMSO_12hpf_whole.jpg]

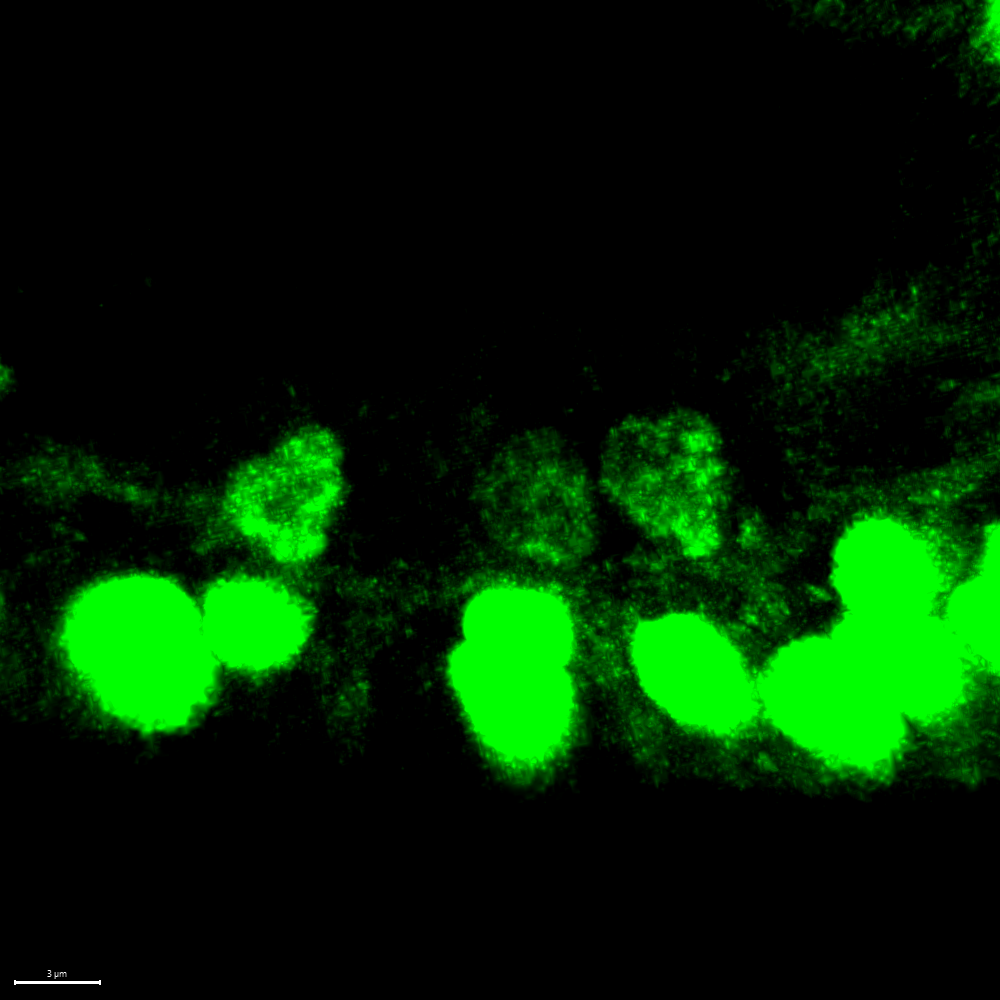

Supplement: Supplementary file 7 — Source data Fig. 4 [file 44319_2024_139_MOESM7_ESM.zip › Figure 4/4B/122620pSer2_inh_[ii8_2_3i_12hpf_4_Image_9]_2020-12-28T11-38-00.799.tif]

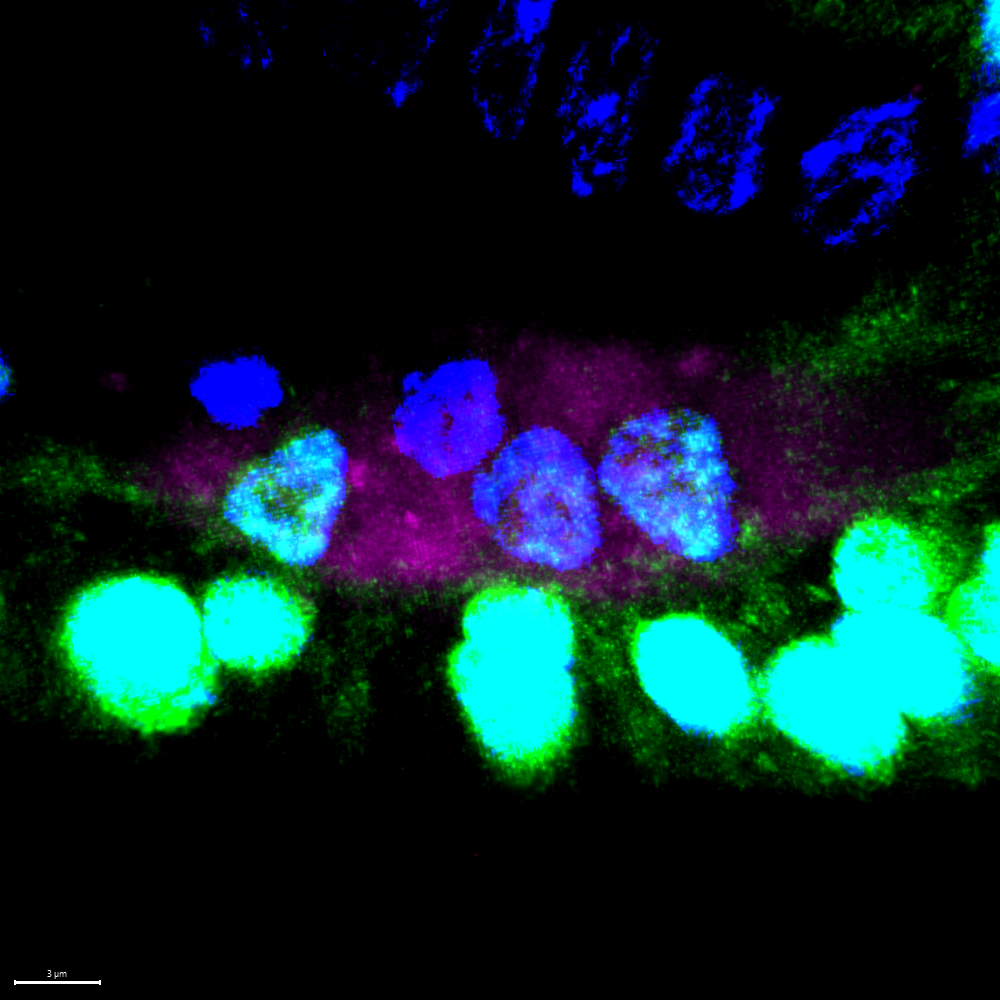

Supplement: Supplementary file 7 — Source data Fig. 4 [file 44319_2024_139_MOESM7_ESM.zip › Figure 4/4B/122620pSer2_inh_[ii8_2_3i_12hpf_4_Image_9]_2020-12-28T11-37-54.655.tif]

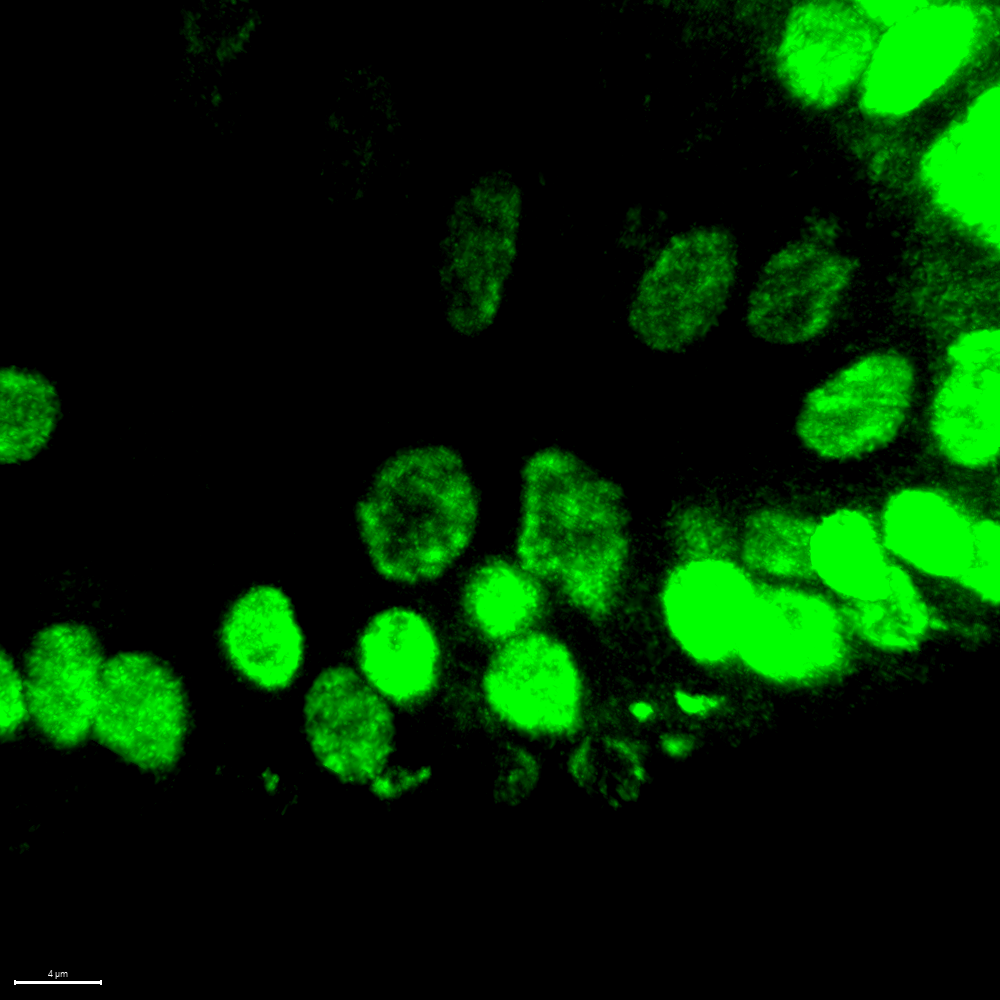

Supplement: Supplementary file 7 — Source data Fig. 4 [file 44319_2024_139_MOESM7_ESM.zip › Figure 4/4C/122620pSer2_inh_[ii16_3_Ruxo_12hpf_6_Image_17]_2020-12-28T11-39-50.191.tif]

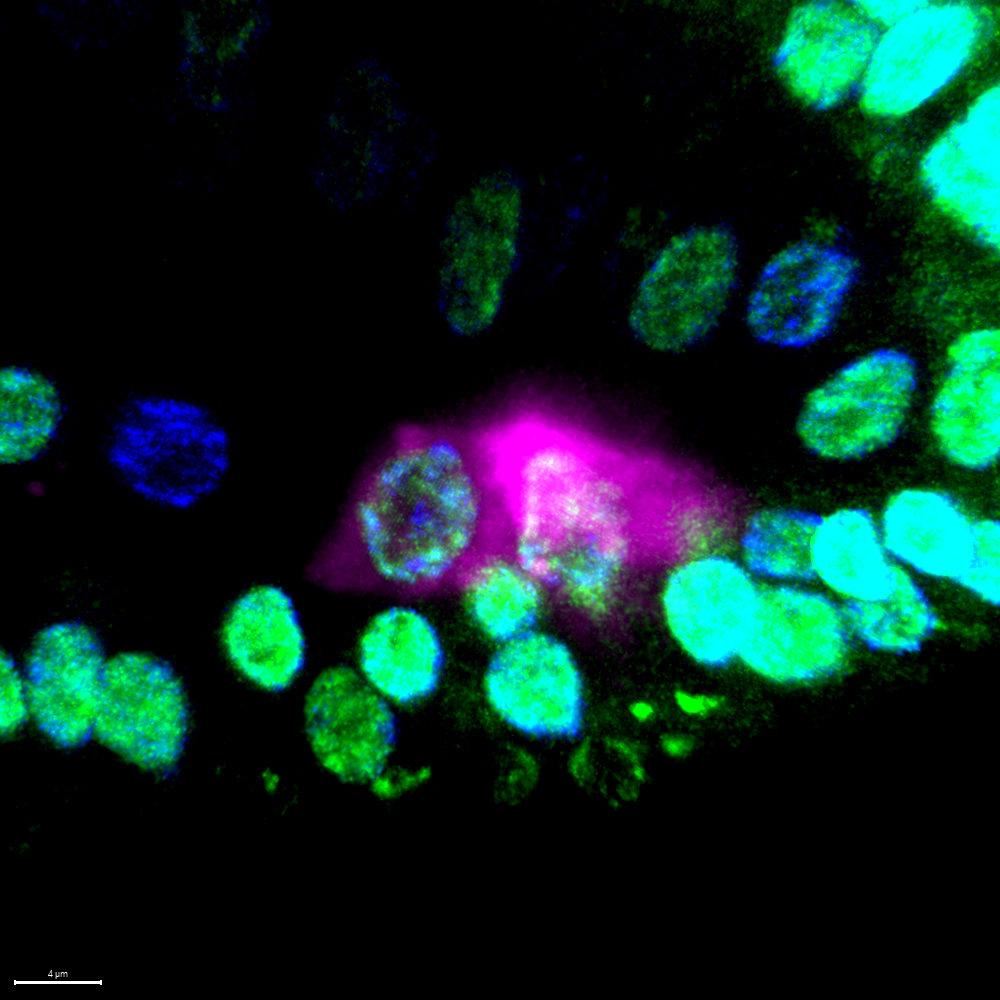

Supplement: Supplementary file 7 — Source data Fig. 4 [file 44319_2024_139_MOESM7_ESM.zip › Figure 4/4C/122620pSer2_inh_[ii16_3_Ruxo_12hpf_6_Image_17]_2020-12-28T11-39-44.196.tif]

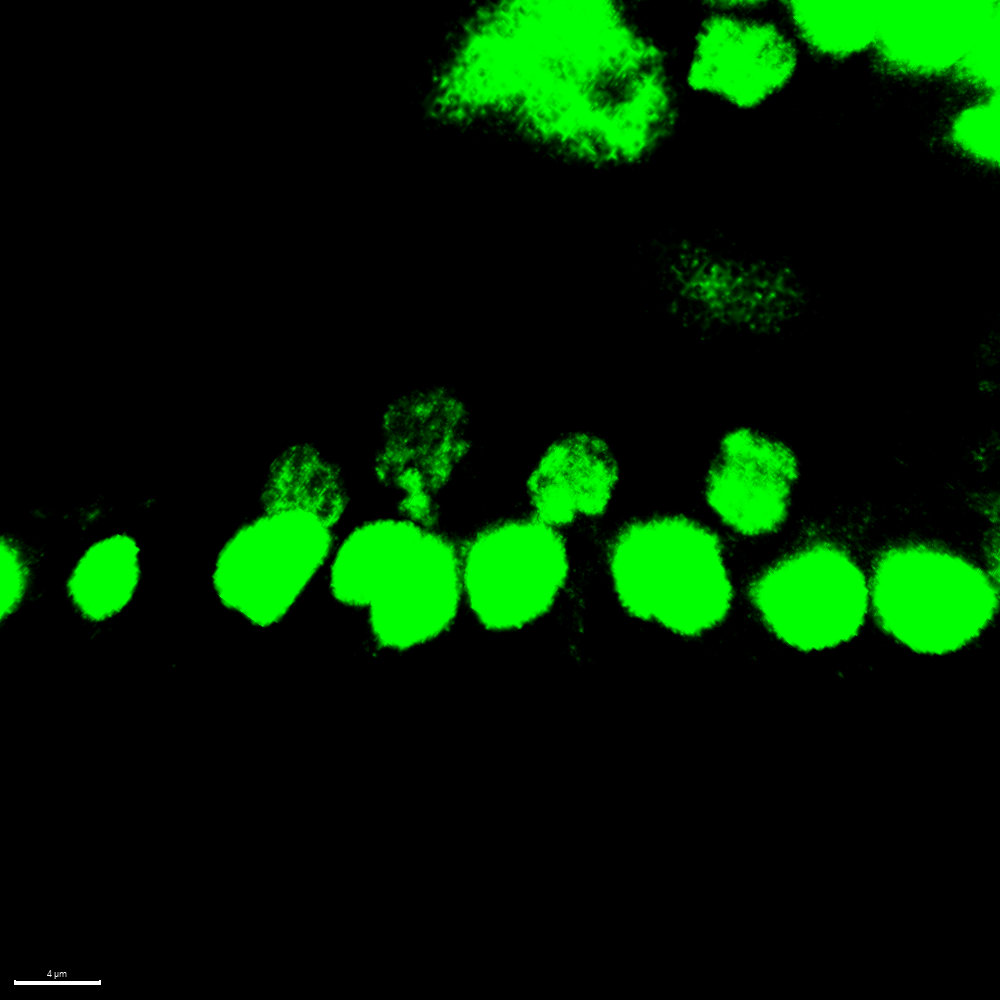

Supplement: Supplementary file 7 — Source data Fig. 4 [file 44319_2024_139_MOESM7_ESM.zip › Figure 4/4A/122620pSer2_inh_[ii2_1_DMSO_12hpf_3_Image_3]_2020-12-28T11-33-37.418.tif]

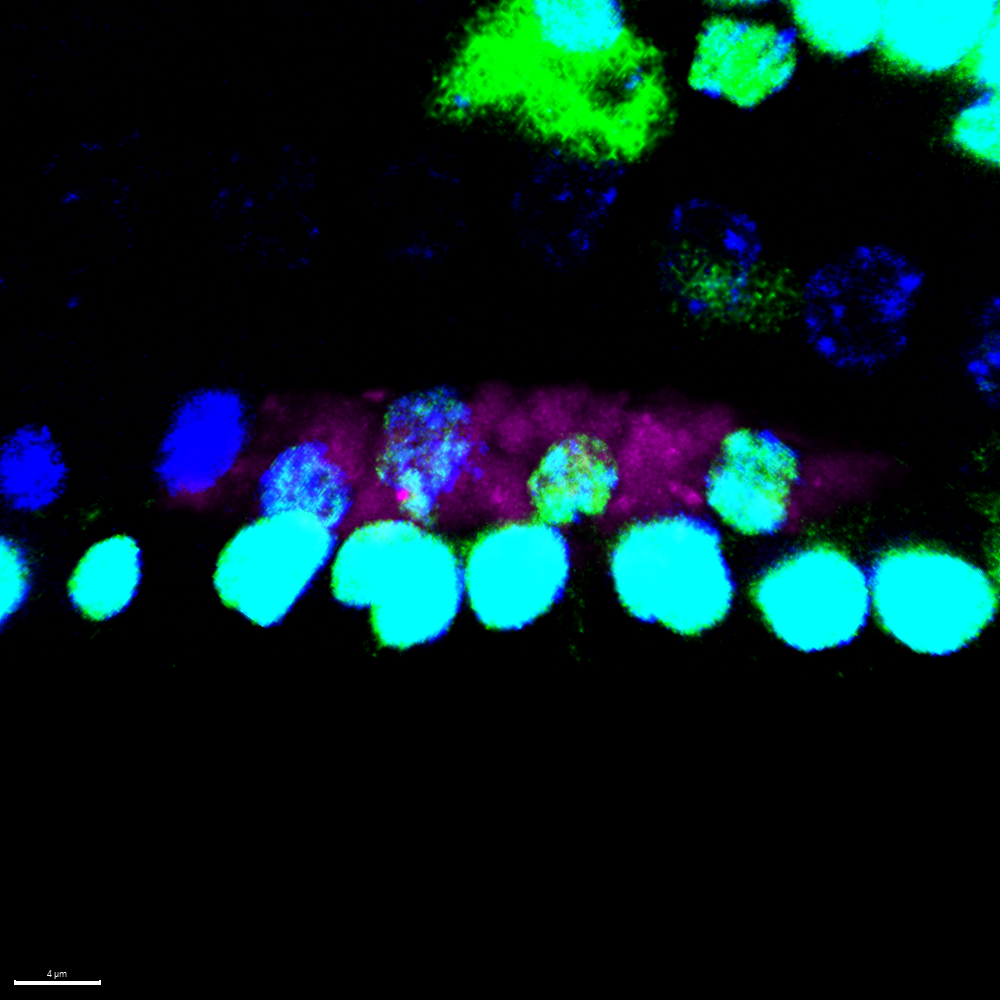

Supplement: Supplementary file 7 — Source data Fig. 4 [file 44319_2024_139_MOESM7_ESM.zip › Figure 4/4A/122620pSer2_inh_[ii2_1_DMSO_12hpf_3_Image_3]_2020-12-28T11-33-28.863.tif]
